# Supplementary material for: Comparison of scenario reduction approaches for reservoir inflow timeseries generated by a Bayesian Neural Network
Source: PLoS One. 2026 May 27;21(5):e0350095. doi: 10.1371/journal.pone.0350095 (PMC13215500; doi:10.1371/journal.pone.0350095)
Supplement: Attachment — Submitted filename: Review_Response_JKoo__PLOS_One.pdf [file pone.0350095.s001.pdf]

Revision Note Introduction

We truly appreciate the feedback of the Editor and Reviewers. Your remarks have allowed us to improve the quality of our manuscript. Below, we explain how the comments and remarks have been addressed in the revised manuscript. To enhance the readability of this response letter, all the Editor's and Reviewers' comments are typeset in blue boxes. Rephrased or added sentences from the revised manuscript are typeset in gray boxes. Note that line numbers refer to the updated version of the manuscript. The respective parts in the revised manuscript are also highlighted to indicate changes. To summarize, the major changes are listed as follows:

1. The abstract has been completely rewritten to improve clarity and readability.
2. The explanation of scenario reduction algorithms and their justification has been added to enhance readers' understanding.
3. In the discussion section, the research scope (target) and the limitations of BNN have been presented in more detail.

Authors' Response to the Editor

Comment 1

Please ensure that your manuscript meets PLOS ONE's style requirements, including those for file naming.

Response: Thank you for this constructive feedback.

We updated the style of our manuscript following PLOS ONE's requirements, e.g., the format of sections and subsections, referencing figures, and so on.

Comment 2

We note that Figure 1 in your submission contains [map/satellite] images, which may be copyrighted. All PLOS content is published under the Creative Commons Attribution License (CC BY 4.0), which means that the manuscript, images, and Supporting Information files will be freely available online, and any third party is permitted to access, download, copy, distribute, and use these materials in any way, even commercially, with proper attribution. For these reasons, we cannot publish previously copyrighted maps or satellite images created using proprietary data, such as Google software (Google Maps, Street View, and Earth).

Response: Thank you for this constructive feedback.

We discovered that one of the base maps, the Digital Elevation Model (DEM), is licensed under CC-BY-NC-ND, which prohibits commercial use. As a result, we revised Fig 1 by removing the base DEM and using only the basin and river shapefiles.

The shapefiles used in Fig 1 can be downloaded from the relevant organizations. Further license information (CC-BY) can be found at the following links:

- River - https://www.vworld.kr/dtmk/dtmk_ntads_s002.do?dsId=30207
- Basin - https://www.data.go.kr/data/15058068/openapi.do?recommendDataYn=Y

Unfortunately, these sites are only available in Korean. However, you can use Google Translate in Chrome to review them.

To reflect this change in the manuscript, we have updated the caption of Fig. 1 as follows:

> **Revised caption for Fig 1**: Location of the Daecheong(DC) reservoir and upstream water level stations. The left map shows the DC basin area. The right figure shows the location of the Daecheong Reservoir and water level stations in the upstream area. The basin and river shape files of Korea are from the public data portal in South Korea (www.data.or.kr) and VWorld (https://www.vworld.kr), respectively. They were originally generated by the National Geographic Information Institute (https://www.ngii.go.kr) and the Han River Flood Control Office(https://www.hrfco.go.kr/).

Authors' Response to Reviewer 1

Comment 1

Rewrite the abstract properly by following structure: Background, Objective, Methods, Results, Conclusion.

Response: Thank you for this valuable comment.

To enhance legibility and clarity, we completely rewrote the abstract following your recommendations, which are to follow the general structure, as below.

Revised Abstract: Uncertainty in future inflows is one of the key factors in reservoir flood control. We can consider this uncertainty explicitly in optimal control of reservoirs using scenario-based stochastic control approaches, where time series of inflows could be generated from probabilistic models representing different futures with their associated likelihoods. It then becomes essential to use a sufficiently large number of uncertain scenarios representing possible future states. Too many scenarios may lead to significant computational complexity for control optimisation, but too few scenarios may become less representative. Therefore, a method for preparing scenarios consists of two parts: scenario generation and reduction. Despite an extensive number of studies on these methodologies in various fields, to the best of our knowledge, there is little research tailored to reservoir inflow properties. In this manuscript, we develop and apply a probabilistic data-driven model, specifically a Bayesian Neural Network (BNN), as a scenario generation method. The BNN model captures the temporal dependence of inflow time series and shows high accuracy, as measured by the Nash-Sutcliffe efficiency Coefficient (NSC) and Root Mean Squared Error (RMSE), for short prediction horizons. Accuracy decreases as the prediction time increases. For scenario reduction, an important element is the distance measure for how representative the reduced set is of future inflows. We evaluate the applicability of four distance measures: the Manhattan distance, the Euclidean distance, the Wasserstein distance, and the

energy distance, which are widely used for scenario reduction in other applications. In terms of preserving the statistical characteristics of the original larger scenario set, we find that the energy distance is the best, followed by the Manhattan and Euclidean distance measures. However, regarding the inclusion of extreme scenarios, which are critical in flood control, the Manhattan and Euclidean distances are found to be the best, as measured by a tailored quantitative index. This index represents the size of the envelope of a scenario set (using l_1-norm), which ensures the reduced sets retain the range of maximum and minimum flows of the original scenario. In addition, the results suggest that reduced scenarios can capture extreme scenarios adequately when $m \geq 30$, where m is the reduced scenario set size. In terms of computational complexity, the energy distance exhibits the highest computational cost, scaling quadratically with m, followed by the Wasserstein distance, which increases approximately linearly with m. Overall, considering the importance of extreme scenarios in flood control and the computational complexity of calculating the energy distance, the Manhattan and Euclidean distances with $m = 30$ can be recommended as practical measures for reducing inflow scenarios.

Comment 2

Avoid lengthy descriptions like "we evaluate the applicability of the four distance measures widely used in the literature."

Response: Thank you for this valuable comment.

To improve readability and clarity, we completely rewrote the abstract, incorporating your suggestion, which emphasizes avoiding lengthy descriptions, as presented in our previous response.

Comment 3

Rewrite the objectives separately and clearly.

Response: Thank you for your constructive comment.

We absolutely agree with your recommendations. Even though there was a simple description of the objectives, it is not enough for readers to understand clearly what our research aims to achieve. Therefore, as you point out, we rewrote the objectives separately and clearly in the Introduction section, as follows.

Revised text L89-99: This study aims to address the following objectives: 1) to develop and validate a data-driven probabilistic approach using Bayesian Neural Networks (BNN) for generating realistic reservoir inflow scenarios that capture the temporal dependence of hydrological time series, and 2) to systematically evaluate and compare the performance of different distance measures (Manhattan, Euclidean, Wasserstein, and energy distance) for reservoir inflow scenario reduction, with particular emphasis on their ability to preserve statistical characteristics and less likely extreme values, and 3) to provide practical recommendations on both the optimal number of reduced scenarios and the most suitable distance measures for reservoir flood control applications, considering the trade-off between computational efficiency, statistical accuracy, and preservation of extreme scenarios.

Comment 4

Focus more on gaps in the hydrological scenario reduction.

Response: Thank you for your constructive suggestion.

We fully agree with your comment. It is very important to make it clear that our study focuses on hydrological scenarios, as you point out. Therefore, we also added some

sentences to mention it explicitly in several places in the Introduction section, as follows, in addition to pointing out this in the objectives, as previously revised text.

added text L72-73: Despite the advances in scenario reduction techniques for various applications, significant gaps remain in the context of hydrological scenario reduction.

added text L85-88: While capturing extreme events is crucial for flood control due to their significant impact on hydrological flood conditions, it has not been fully explored which reduction technique best preserves the extreme events from the original scenarios.

Comment 5

Add a clear novelty statement like "To the best of our knowledge, ...

Response: Thank you for your helpful comment.

Although some novelties are described in the newly revised objectives of our research, it is worthwhile to mention them directly, as you point out. Therefore, we added a sentence based on your comment, as follows.

added text L99-102: To the best of our knowledge, this is the first comprehensive study that integrates a verification of a probabilistic data-driven approach for scenario generation and a rigorous evaluation of scenario reduction techniques for hydrological scenarios, especially considering flood scenarios.

Comment 6

Clarify the hyperparameter optimization process. Don't just say TPE is used.

Response: Thank you for this valuable comment.

We fully agree with you. It is important to give some explanation on the algorithm we utilized, even though the implementation of the TPE algorithm is very simple, while it has a sophisticated mathematical background. Following your suggestion, we have added explanations of how the algorithm works and included information about the package we used with its corresponding reference.

We also recognized that our manuscript lacked clarity regarding the validation data composition and the performance metric used for hyperparameter optimization. We have now included additional sentences to address these important details.

Added text L248-249: For the validation, 20% of the data is randomly selected.

Added text L263-273: The Tree-structured Parzen Estimator (TPE) algorithm is a type of Bayesian optimization that models the conditional probability distributions of hyperparameters. In detail, it constructs two density distributions using Kernel Density Estimators, especially Parzen estimators here, as the surrogate models of the hyperparameter distributions associated with superior performance($l(x)$) and inferior performance($g(x)$), where x represents a hyperparameter set. The algorithm iteratively selects new hyperparameter sets by maximizing the expected improvement criterion, defined as the ratio $l(x)/g(x)$, thereby efficiently exploring the search space to identify optimal configurations. Here, the Root Mean Square Error (RMSE) of validation data is utilized as a performance index of hyperparameter sets. We implement this algorithm using Optuna (Akiba et al. 2019), a Python library.

Comment 7

No proper discussion on missing data handling or data cleaning steps.

Response: Thank you for this constructive feedback.

In fact, we did not clean the data because we only used quality-controlled data, which was officially published by the managing organizations. Unlike reservoir data, which contained no missing data or outliers, official water level data from some stations contained missing data and outliers. This is reasonable because data quality regulations for reservoirs do not allow missing data, but for water level stations, data with missing values or outliers are allowed to be published even when they represent actual observations. Instead of cleaning the data ourselves, we excluded those stations entirely. We agree that this requires further explanation, so we have added the following sentences in the revised manuscript.

Added text L224-225: This officially published data has no missing values or outliers since K-water has cleaned the data and checked its precision.

Added text L232-241: Other stations were either installed after 2011 or have missing data even in their officially published records. Unlike reservoir data, which contained no missing data or outliers, official water level data can contain missing data and outliers. This is reasonable because data quality regulations for reservoirs do not allow missing data, but for water level stations, data with missing values or outliers are allowed to be published even when they represent actual observations. Reservoir data are more strictly managed due to both their importance and the straightforward data production process. Further information is available in (Jeong et al. 2021). In this study, instead of cleaning unofficial data ourselves, we only utilize data from stations without missing values or outliers.

Comment 8

Merge figures or reduce redundancy.

Response: Thank you for this constructive comment.

As you pointed out, some of the figures contain redundancy, and many include multiple subfigures. For example, Figures 6 and 9 each have 24 subfigures to display information for all cases and distance measures. Additionally, Figure 8 presents similar information in two ways: the original values and the mean absolute error (MAE). It is clear that these figures exhibit redundancy, as you mentioned. However, our intention was to illustrate the differences between each distance measure more specifically. For instance, Figures 6 and 9 visually demonstrate that our results do not vary depending on the case. Furthermore, in Figure 8, the meaning of the MAE differences can be understood through the original values. Specifically, for the Manhattan and Euclidean distances, where the MAE for mean and standard deviation is relatively large compared to other measures, the relative MAE is not as large. However, if we provide only figures showing the relative MAE values, these values and their differences are too small to clearly illustrate the advantages and disadvantages of the distance measures.

Although there is some redundancy, we kindly ask for your understanding that the inclusion of these figures was intentional. If there are no strict limitations regarding the overall length of the paper or the number of figures, we would appreciate your understanding. However, if you believe that the redundancy in the figures may hinder the reader's understanding, we would be grateful for more specific feedback, and we are fully prepared to make any necessary revisions.

Comment 9

In Discussion, there is no critical comparison with past scenario reduction studies.

Response: Thank you for your insightful comment.

Regarding the comparison with past scenario reduction studies, as we added in the Introduction following your suggestion, like 'this is the first study that applies scenario reduction techniques in hydrological scenarios.' Moreover, this study aims to evaluate the pros and cons of existing methodologies from the perspective of hydrological scenarios, instead of developing new methodologies. However, we agree that readers can wonder why the comparison is missing, as you point out. Therefore, we added some sentences to clarify this, as follows.

> **Added text L405-408**: Our research aims to verify the applicability of probabilistic data-driven models to generate uncertain scenarios considering the characteristics of hydrological scenarios. Here, the characteristics include both the temporal correlations and the accuracy degradation with increasing the prediction horizon.

> **Added text L427-430**: We evaluate the advantages and disadvantages of existing scenario reduction methodologies, particularly from the perspective of distance measures. The performances are assessed by three criteria: preservation of statistical properties, inclusion of extreme events, and computational complexity.

Comment 10

In Discussion, discuss more about the limitations of BNN for peak inflow predictions.

Response: Thank you for your thoughtful comment.

Regarding the BNN's limitation in peak inflow predictions, it is clearly caused by the imbalanced training data. Naturally, there are more low-inflow data than high or peak-inflow data, even when considering only flood events. However, due to the lack of data, we utilized all hourly data, including drought seasons. This leads to shifting the probability of peak inflow toward the extreme tail. It is important to explain this in more detail, as you pointed out. We have added the following explanation.

Added text L417-426: Extreme values, such as peak flood inflow, are located at the tail of the data distribution, making accurate prediction difficult. When the model is trained predominantly on lower inflow values, it struggles to predict extreme values, leading to overfitting on small inflows and underfitting on extreme events (Shi et al. 2024; Ding et al. 2019). We use all hourly data from 2012 to 2020 to train the BNN model. There are only one or two flood events each year, and there are only nine flood events during this period, as described in Section 2.1. This implies that most training data does not have considerable inflows. In addition, in most cases, the inflow decreases very soon without demonstrating significant increases, even when the inflow increases. Therefore, the model's inability to predict peak values can be attributed to insufficient exposure to extreme data.

Comment 11

In Discussion, add a clear recommendation for practitioners on when to use which distance measure.

Response: Thank you for your valuable comment.

We only presented the advantages of the Euclidean and Manhattan distances in our original manuscript. However, the energy distance significantly outperforms other distance measures in terms of preserving statistical properties. Therefore, it seems better to clearly point out when those measures are advantageous, as you recommend. Moreover, how many scenarios are appropriate for hydrological scenario reduction is added here again, as follows.

Added text L458-463: However, when preserving the statistical properties, such as mean, standard deviation, and temporal correlation, is prioritized over including extreme scenarios, and computational time is not a critical concern, the energy

distance can be the best choice. Regardless of the distance measure selected, reducing the number of scenarios to 30 is sufficient in terms of both inclusion of extreme events and preservation of statistical properties.

Comment 12

Rewrite long sentences or maybe split into two clear sentences.

Response: Thank you for your constructive feedback.

We have also split long sentences into two short sentences, following your suggestion, like in lines 155-158, 303-305, 325-328, and 409-413, throughout the entire paper.

Comment 13

Read these studies to further enrich your study. (DOI: 10.1016/j.asoc.2023.110722) Provides a soft computing approach for hydrological prediction, which can complement BNN in uncertainty modeling. (DOI: 10.1007/s11269-025-04142-5) Demonstrates different ML models for water prediction, useful to compare or benchmark scenario generation models. (DOI: 10.2166/wpt.2024.147) Discusses ML-based hydrological modeling that can supplement your scenario generation techniques. (DOI: 10.5772/intechopen.1006491) Discusses novel fuzzy-based models that can be integrated or compared with BNN-based scenario reduction. These studies are suggested for you to check the content and improve your paper. IF THEY ARE APPROPRIATE ONLY.

Response: Thank you for this valuable suggestion.

The studies you suggested can provide valuable insights for future research and enrich our understanding of data-driven approaches for predicting hydrological variables. We

especially think that the Cascaded-ANFIS model, which showed significant performance in simulating rainfall-runoff, can be a perfect deterministic model for our ongoing study. As we mentioned in the Conclusion, we plan to compare a stochastic data-driven approach with a data-driven deterministic approach. We believe that there are pros and cons to each approach, and hopefully present them. Thank you again for these references.

Authors' Response to Reviewer 2

General Comments. To further strengthen the manuscript, we suggest a few improvements. First, while the authors have described the data sources and provided processed datasets, a more detailed explanation of the data preprocessing steps—such as how missing values and anomalies were handled—would enhance the reproducibility and transparency of the study. Second, although the comparison of scenario reduction methods is comprehensive, the current setup associates each distance metric with a different reduction algorithm (e.g., clustering for Manhattan and Euclidean distances, forward selection for Wasserstein and energy distances). To isolate the pure effect of the distance metrics, it would be valuable to apply a uniform reduction algorithm across all metrics. Third, while the study clearly recommends practical distance measures considering computational constraints, it could further benefit practitioners by providing specific operational guidelines, such as recommended numbers of reduced scenarios under given time limits. Overall, these additions would make the already strong manuscript even more robust and practically impactful.

Response: We sincerely appreciate your thoughtful review. All concerns have been carefully considered and addressed in our revised manuscript, as outlined below. These comments have significantly helped us to improve the quality of our manuscript.

Comment 1

First, while the authors have described the data sources and provided processed datasets, a more detailed explanation of the data preprocessing steps—such as how missing values and anomalies were handled—would enhance the reproducibility and transparency of the study.

Response: Thank you for this valuable comment.

The data we used is already quality-controlled data, which was officially published by the managing organizations. Therefore, there are no missing values or outliers in the reservoir data. However, official water level data from some stations contained missing data and outliers. This is reasonable because data quality regulations for reservoirs do not allow missing data, but for water level stations, data with missing values or outliers are allowed to be published even when they represent actual observations. Instead of preprocessing the data ourselves, we excluded those stations entirely. Even though we provided all data directly downloaded from official websites of South Korean organizations as described in the acknowledgments section, we realized that this requires further explanation. We have added the following sentences to the revised manuscript.

Added text L224-225: This officially published data has no missing or outlier data since K-water has cleaned the data and checked its precision.

Added text L232-241: Other stations were either installed after 2011 or have missing data even in their officially published records. Unlike reservoir data, which contained no missing data or outliers, official water level data can contain missing data and outliers. This is reasonable because data quality regulations for reservoirs do not allow missing data, but for water level stations, data with missing values or outliers are allowed to be published even when they represent actual observations. Reservoir data are more strictly managed due to both their importance and the straightforward data production process. Further information is available in Jeong et al. (2021). In this study, instead of cleaning unofficial data ourselves, we only utilize data from stations without missing values or outliers.

Comment 2

Second, although the comparison of scenario reduction methods is comprehensive, the current setup associates each distance metric with a different reduction algorithm (e.g., clustering for Manhattan and Euclidean distances, forward selection for Wasserstein and energy distances). To isolate the pure effect of the distance metrics, it would be valuable to apply a uniform reduction algorithm across all metrics.

Response: Thank you for this constructive comment.

We believe this comment is very important. As you point out, we obviously utilized different algorithms for different distance measures, except for the Wasserstein and energy distances.

The different properties of each distance measure cause this. The Manhattan and Euclidean distances are the distances between vectors, i.e., each scenario. However, the Wasserstein and energy distances can be measured between probability distributions, i.e., a distribution of original scenarios and a distribution of reduced scenarios. This difference leads to different algorithms. We can use the distances between two scenarios to sort scenarios, i.e., clustering, and pick one representative scenario in each cluster. On the contrary, we can compare the similarity between the original scenario set and the reduced set using the Wasserstein or energy distance. Thus, we can add a scenario to the reduced set, which starts with zero elements, one by one, and check if the added scenario is the best in terms of distances between two distributions (forward selection). Also, we can subtract a scenario from the reduced set, which originally includes all original scenarios.

Suppose we apply the forward selection or backward selection method to the Manhattan or Euclidean distance. In that case, it is obvious that only the scenario closest to the mean is selected at every step. Furthermore, it is not possible to apply any clustering algorithms using the Wasserstein or energy distance. Therefore, it looks obvious that we do not have diverse choices for algorithms if we have already decided on a distance

measure. In addition, if we can not apply the same algorithm for all distance measures, it would be better to apply the ‘best’ or ‘widely-used’ algorithm in order to compare the results fairly. That is why we utilize the K-means for the Euclidean, K-median for the Manhattan, and forward selection for the Wasserstein and energy distance.

However, we found that the detailed explanation is omitted from our original manuscript. To enhance clarity, we have added a detailed explanation, as below.

Added text L194-217: It is worthwhile to clarify that different algorithms are utilized for different distance measures. This is due to the different properties of each distance measure. The Manhattan and Euclidean distances are the distances between vectors, i.e., each scenario. However, the Wasserstein and energy distances can be measured between probability distributions, i.e., a distribution of original scenarios and a distribution of reduced scenarios. This difference leads to the utilization of different algorithms. We can use the distances between two scenarios to sort scenarios, i.e., clustering, and pick one representative scenario in each cluster. In contrast, we can compare the similarity between the original scenario set and the reduced set using the Wasserstein or energy distance (Heitsch et al. 2007; Ziel 2021). Thus, we can either add scenarios to the reduced set (which starts empty) one by one, selecting the scenario that minimizes the distance between the two distributions (forward selection), or subtract scenarios from the reduced set (which initially includes all original scenarios). Alternatively, the reduced scenario set can be found directly by calculating all possible combinations of reduced scenarios and selecting the one with the smallest distance from the original scenarios.

If we apply the forward or backward selection method to the Manhattan or Euclidean distance, it is clear that only the scenario closest to the mean would be selected at each step. Furthermore, it is not possible to apply any clustering algorithms using the Wasserstein or energy distance. Therefore, it is evident that we only have limited feasible algorithms once a distance measure is selected. In addition, given that applying the same algorithm for all distance measures is not

possible, it would be better to apply the ‘best’ or ‘widely-used’ algorithm for each distance measure to ensure a fair comparison. This is why we utilize the K-means for the Euclidean, K-median for the Manhattan, and forward selection for the Wasserstein and energy distance.

In addition, to maintain consistency, we revised that describe the limitation of the scenario reduction approach here, as below.

Revised text L464-473: It should be noted that we employ a simple 1-step forward selection method for the Wasserstein and energy distances. Even though it has been widely applied due to its computational efficiency, this algorithm can not guarantee the selection of the optimal reduced set. To find the optimal reduced set closest to the original scenario set, all possible subsets should be explored. This is generally computationally intractable. However, the implementation of high-performance computing and/or parallelization techniques could potentially allow us to obtain the optimal reduced set based on the energy or Wasserstein distance with significantly reduced computational time. It remains to be determined whether this algorithm can improve the performance of these distance measures and whether reduced computational time is practical.

Comment 3

Third, while the study clearly recommends practical distance measures considering computational constraints, it could further benefit practitioners by providing specific operational guidelines, such as recommended numbers of reduced scenarios under given time limits.

Response: Thank you for this constructive comment.

You are correct. Even though we mentioned that $m = 30$ is sufficient, where the number of

reduced scenarios is m, in section 2.2, it is worthwhile noting this again in the Discussion section. In addition, we only present the advantages of the Euclidean and Manhattan distances. However, the energy distance significantly outperforms other distance measures in terms of preserving statistical properties. Therefore, it seems better to clearly point out when those measures are advantageous, as you recommend, as follows.

Revised and Added text L456-463: Consequently, considering the importance of extreme scenarios for optimal flood operation, the Manhattan and Euclidean distances can be reasonable and practical choices for scenario reduction. However, when preserving the statistical properties, such as mean, standard deviation, and temporal correlation, is prioritized over including extreme scenarios, and computational time is not a critical concern, the energy distance can be the best choice. Regardless of the distance measure selected, 30 reduced scenarios can be sufficient in terms of both inclusion of extreme events and preservation of statistical properties.

References

Akiba, Takuya, Shotaro Sano, Toshihiko Yanase, Takeru Ohta, and Masanori Koyama. 2019. "Optuna: A Next-generation Hyperparameter Optimization Framework." In *Proceedings of the 25th ACM SIGKDD International Conference on Knowledge Discovery and Data Mining.*

Ding, Daizong, Mi Zhang, Xudong Pan, Min Yang, and Xiangnan He. 2019. "Modeling extreme events in time series prediction." In *Proceedings of the 25th ACM SIGKDD International Conference on Knowledge Discovery & Data Mining,* 1114–1122.

Heitsch, Holger, and Werner Römisch. 2007. "A note on scenario reduction for two-stage stochastic programs." *Operations Research Letters* 35 (6): 731–738.

Jeong, Gimoon, Do-Guen Yoo, Tae-Woong Kim, Jin-Young Lee, Joon-Woo Noh, and Doosun Kang. 2021. "Integrated quality control process for hydrological database: a case study of Daecheong Dam basin in South Korea." *Water* 13 (20): 2820.

Shi, Jimeng, Azam Shirali, and Giri Narasimhan. 2024. "Boosting Time Series Prediction of Extreme Events by Reweighting and Fine-tuning." In *2024 IEEE International Conference on Big Data (BigData),* 1450–1457. IEEE.

Ziel, Florian. 2021. "The energy distance for ensemble and scenario reduction." *Philosophical Transactions of the Royal Society A* 379 (2202): 20190431.
