# Supplementary material for: Comparison of scenario reduction approaches for reservoir inflow timeseries generated by a Bayesian Neural Network
Source: PLoS One. 2026 May 27;21(5):e0350095. doi: 10.1371/journal.pone.0350095 (PMC13215500; doi:10.1371/journal.pone.0350095)
Supplement: Attachment — Submitted filename: Response to Reviewers_JKoo_R2.pdf [file pone.0350095.s002.pdf]

**Revision Note Introduction**

Dear Editor and Reviewers,

We truly appreciate your feedback and suggestions, which we believe have allowed us to improve the quality and readability of the manuscript. Below, we explain how the comments and remarks have been addressed in the revised manuscript. To enhance the readability of this response letter, all the Editor's and Reviewers' comments are typeset in blue boxes. Rephrased or added sentences from the revised manuscript are typeset in gray boxes. Note that line numbers, figures, and sections in the response refer to the updated version of the manuscript. The respective parts in the revised manuscript are also highlighted to indicate changes. To summarize, the major changes are listed as follows:

1. A flowchart has been added to the Method section (Fig 1) to illustrate the research process more clearly.
2. The limitations of the study, including the exclusion of numerical experiments using stochastic optimization-based feedback control and the inaccuracy of BNN models in predicting peak inflows, have been presented and explained.
3. The abstract and conclusion have been carefully rewritten to improve clarity and readability by making them more concise and reducing redundancy.

In addition to the modifications made in response to the editor's and reviewers' comments, several sentences have been revised to improve clarity and readability, without altering their meaning.

# Authors' Response to the Editor

**Comment 1**

Add a flowchart in the methodology section to illustrate the workflow of the current study.

**Response:** Thank you for this constructive feedback.

We agree with your suggestion and have added the Flowchart shown below (Figure 1), illustrating our methodologies and described at the end of the Method section, as follows:

**Added text L229-233**: The method presented in this section is illustrated in Fig 1. Scenario generation is performed using MC dropout BNN, and the resulting (very large number of) scenarios are reduced using four distance measures. These reduced scenarios are then evaluated based on criteria that best replicate the original generated scenarios. The details of this process are provided in the following sections.

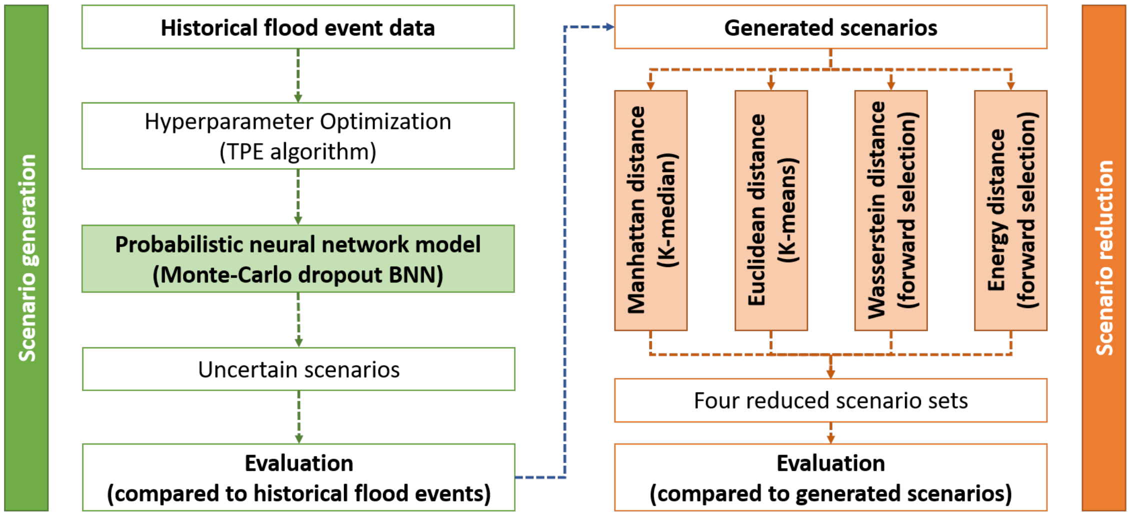


Figure 1: Flowchart of the study framework, comprising scenario generation and scenario reduction. The methods are applied to reservoir flood inflow time series to assess their practical applicability.

**Comment 2**

Ensure that each table and figure is clearly explained and discussed in the main text. The values presented in the tables should be mentioned and discussed in the text. Figures also require more detailed discussion in the text.

**Response:** Thank you for this valuable comments.

We added some explanatory sentences for some figures and tables in the main text as follows:

**Revised and added text for Table 1 L291-295**: The optimal hyperparameters are presented in Table 1. The optimal autoregressive horizon is 24, resulting in a total of $24 \times 18$ input nodes. The optimal dropout rate is 10%, with 512 nodes in the hidden layer, a learning rate of 0.0005, and a batch size of 64. The selected activation function is the Rectified Linear Unit (ReLU). The architecture of the BNN is illustrated in Fig 3.

**Revised and added text for Fig 9 L385-389**: For example, in Case 3, the envelope volumes obtained using the Manhattan and Euclidean distances are approximately 2.5 $10^3 m^3/s$, which are significantly larger than those obtained using the Wasserstein and energy distances, both of which are less than 1.5 $10^3 m^3/s$, when $m = 10$.

**Revised and added text for Fig 10 L404-407**: Regarding scenario means, the energy distance demonstrates the closest alignment with the original scenarios and the lowest Mean Absolute Errors (MAE) of means in Fig **??**b, which is 2.1 $m^3/s$ when $m = 10$ and decreases to 0.4 $m^3/s$ when $m \geq 40$.

**Revised and added text for Fig 10 L415-416**: For instance, the MAE of the standard deviations of the Wasserstein distance decreases from 11.6 $m^3/s$ to 3.8

$m^3/s$.

**Revised and added text for Table 2 L439-442**: The computational time increases linearly as the number of reduced scenarios and/or original scenarios increases, from 11.8 seconds to 82.3 seconds for the Wasserstein distance and from 72.5 to 1,787.3 seconds for the energy distance.

**Revised and added text for Table 2 L446-449**: For the Manhattan and Euclidean distances, the computation time remains relatively constant regardless of increases in $m$, around 4 seconds for the Manhattan distance and 4.5 seconds for the Euclidean distance, as demonstrated in Table 2.

# Authors' Response to Reviewer 2

**General Comments.** Although the contribution is significant, the evidence supporting the claims is weak and the conclusions are currently unconvincing, so more substantial revisions are needed.

**Response:** We thank to your constructive review. As shown below, we have carefully considered and addressed all points raised. The remarks have allowed us to improve the quality of the manuscript.

**Comment 1**

There have been no cases in which the generated and reduced scenarios have been applied to actual stochastic optimal control, and their actual effectiveness has not been fully proven. For this reason, it is unclear whether the proposed method will directly lead to improved operational performance. Related papers must be cited to make claims.

**Response:** Thank you for this valuable comment.

In general, scenario generation and reduction methodologies are developed to support stochastic optimization approaches. As such, it is common to evaluate their effectiveness and efficiency by applying them to stochastic optimal control problems. One reason for this is the lack of a clear relationship between scenario uncertainty and the performance of stochastic optimal control. Therefore, numerical experiments on stochastic optimal control at least are necessary to verify those methodologies.

However, we consider stochastic optimal control to be beyond the scope of this paper. Incorporating it would require substantial additional work, such as defining specific objectives and constraints. Nevertheless, our subsequent study is to develop and evaluate stochastic optimal control approaches for reservoir flood control, using the scenario generation and reduction techniques proposed in this study.

In our following study, we are adopting a stochastic control approach to verify the effectiveness of scenario generation and reduction methodologies presented in this research. As shown in Fig 2, which is from our ongoing study, Stochastic Model Predictive Control (SMPC) outperforms Deterministic Model Predictive Control (DMPC) in terms of maintaining Reservoir Water Level (RWL) within thresholds. For example, SMPC does not violate the lower threshold and violates the upper threshold less often DMPC.

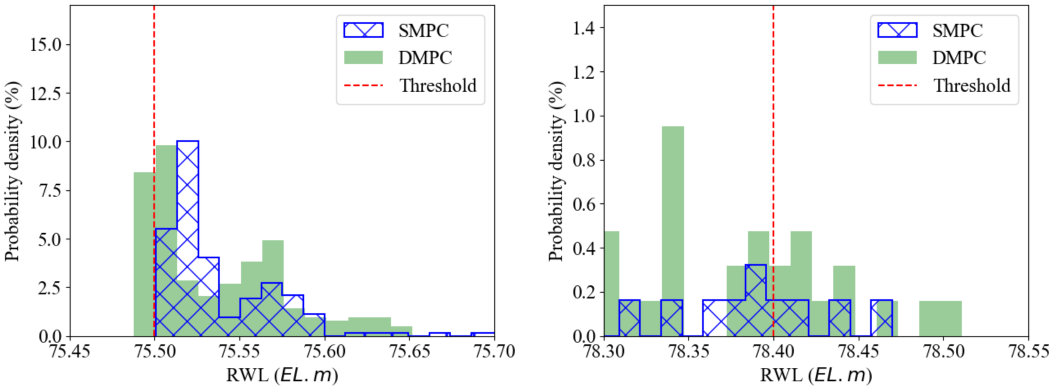


(a) Zoomed-in view around $S_L$

(b) Zoomed-in view around $S_H$

Figure 2: Distribution of RWLs of observation, DMPC, and SMPC.

Additionally, we had compared the results from different distance measures for scenario reduction, for example, the Euclidean distance and energy distance. One of the comparison results is illustrated in Fig 3. In these assessments, we found that the Euclidean distance shows a marginally better and often similar performance compared to the energy distance in constraint violations. Therefore, the conclusion was that the computationally cheap Euclidean distance performs similarly to energy distance-based reduction, and so would be favoured for stochastic MPC approaches.

Even though other factors may affect those results, e.g., the objectives and constraints of a stochastic problem, the way to adopt uncertain scenarios, we believe that those results imply the effectiveness of scenario generation and reduction methodologies we discussed in this research.

Another important reason why our primary focus is on scenario generation and reduction methodologies is that there are broader applications involving uncertain scenario

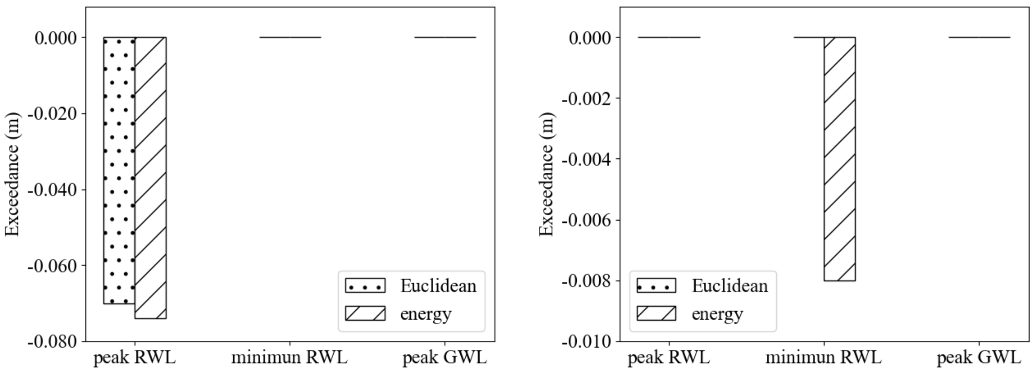


(a) Event 1 (b) Event 2

Figure 3: Results showing distributions of RWL and constraint violations when two different scenario reduction measures are used.

generation and reduction. For example, our approach is relevant to simulation-based methods, which benefit from a reduced set of representative scenarios. In South Korea and many other countries, organizations in charge of reservoir operation still adopt a simulation-based control approach. Operators (or weather agencies) generate many uncertain inflow (or rainfall) scenarios. Operators then generate a best control schedule for each inflow scenario using reservoir simulation tools. Therefore, they have many probable schedules and assess them to decide on a final control schedule through trial and error. The scenario generation and reduction methodologies also benefit those operators because these methodologies can provide a limited number of uncertain scenarios covering extreme events. Therefore, we believe this research contributes meaningfully by presenting and evaluating scenario generation and reduction methods tailored to reservoir flood inflow forecasting.

**Comment 2**

The accuracy of peak prediction in real data is clearly low, raising doubts about the reliability of BNN in flood response. This is due not only to the limitations of the model but also to bias in the training data, so data selection and pretreatment must be reconsidered.

**Response:** Thank you for this constructive feedback.

We agree with your opinion and recognise those. In general, it is challenging to predict peak inflows and other flood inflows accurately simultaneously. Therefore, many studies that utilized data-driven models to predict future inflow hydrographs focused only on one of them. They showed reliable performances from their perspective, but generally we cannot say that their peak predictions outperformed ours (e.g., W. Li et al. 2021; Wang et al. 2025; Liu et al. 2020). We have added literature to reflect this limitation,

**Added text L312-313**: This issue is also evident in several studies on flood inflow prediction, as considerable errors can be observed in their results (e.g., [52-54]).

The degradation of the peak prediction is mainly due to the limitation of training data, i.e., the lack of sufficient data with high inflow. We need to utilize the data from the entire period for model training. However, the inflow changes very smoothly during the majority of the training period. It means that most peaks in the hydrograph are significantly small compared to the peaks of flood events, which we are concerned about. To clearly describe this, we revised a paragraph as follows:

**Revised text L319-327**: This degradation is mainly attributed to the limitation of training data, i.e., the lack of sufficient data with high inflow. While we utilize the data from the entire period for model training, the majority of cases have peaks that are much smaller than shown for Event 1 and Event 2 in Fig 5. Typical inflow patterns feature a modest rise in upstream water level and inflow for a brief period, followed by a gradual decline.

Training the BNN model exclusively on considerable flood events, such as our test events, could enhance prediction accuracy for those extremes. However, due to the small sample size, this would substantially reduce overall model performance and compromise its practical applicability.

Moreover, at least one of the scenarios generated by our BNN models is likely to predict the extreme cases, i.e., the peaks, accurately, thanks to the characteristics of probabilistic approaches, even though the average of scenarios cannot capture the peaks in some cases, e.g., Event 2 in our study. To explain and show this advantage of a BNN model, we have added a paragraph and a figure as follows:

**Added text L328-336**: Despite the limitations in peak inflow prediction, a key advantage of probabilistic approaches is their ability to capture a wide range of uncertainty. For example, the maximum inflows among all scenarios for Events 1 and 2 are 4,393.9 $m^3/s$ and 3,065.2 $m^3/s$, respectively. This indicates that, although the predicted peak inflow (i.e., the mean of the scenarios)—which would be the output of a typical deterministic model—is substantially lower than the observed peak, the actual inflow values fall within or near the upper bound of the predicted range. This is illustrated in Fig 6 and highlights the importance of retaining extreme scenarios, even when they have low likelihoods, to effectively capture peak inflows during flood events.

In addition, we have added a sentence to clearly describe the differences between the previous figure (Fig. 5) and the current one (Fig. 6), in order to prevent possible misunderstanding by readers, as follows:

**Added text to the caption of Fig 5**: he red lines indicate the scenario means of predictions for the next 12 hours, updated every hour.

we have added a discussion on this in the Discussion section, as follows:

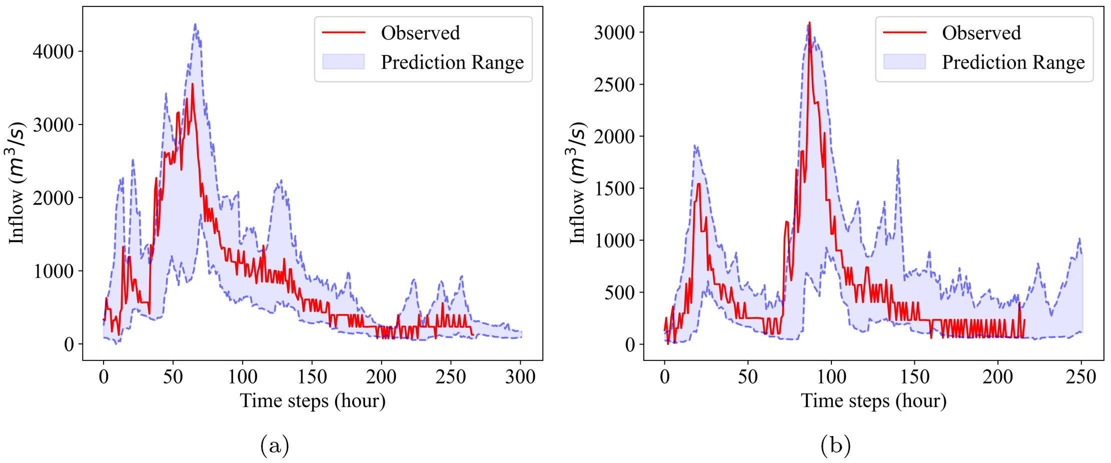


Figure 6: Real inflows and prediction ranges of BNN models for test events: (a) Event 1 (b) Event 2. Prediction ranges are defined by the maximum and minimum values across predicted scenarios, generated every hour for the next 12 hours.

**Added text L472-476**: Despite the limitations in peak prediction, our results demonstrate that at least one of the generated scenarios captures the actual peak, owing to the inherent characteristics of probabilistic approaches. This observation motivates the evaluation criteria for scenario reduction methods, specifically, whether a given method can preserve extreme events in the reduced scenario set (Fig 6).

It is important to note that our BNN model can predict peaks quite accurately for other cases in our following study, as follows (Note that these graphs are also the averages of all scenarios for events.):

Even though we do not evaluate the accuracy of the peaks, it looks like a BNN model can predict the peak in general, except for highly fluctuating cases or extremely narrow peaks, as shown in Fig 7 and Fig 5 in our original manuscript.

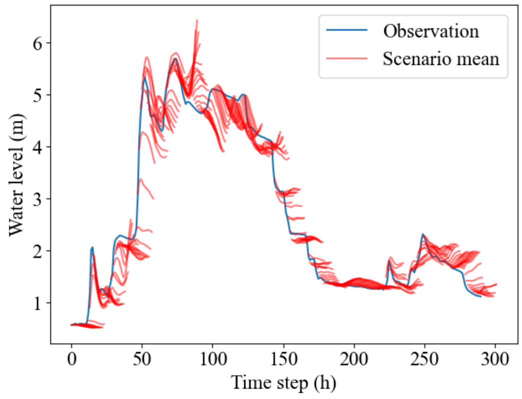


(a) GP water level for event 1

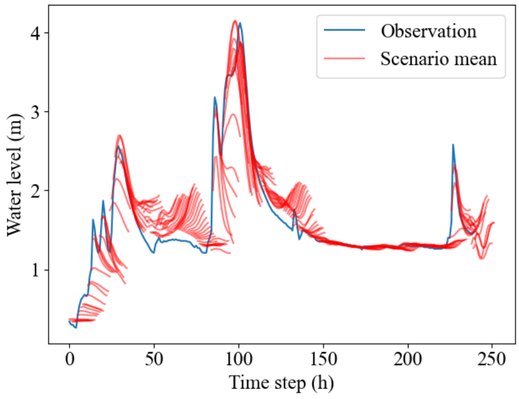


(b) GP water level for event 2

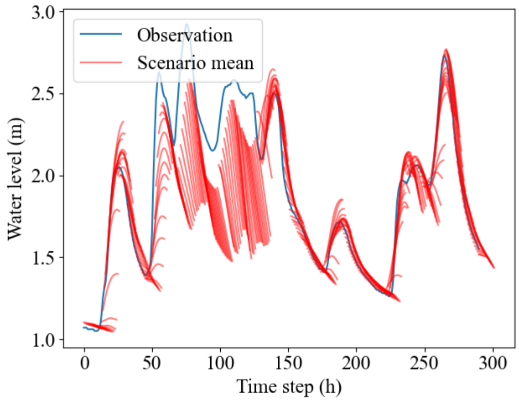


(c) MH water level for event 1

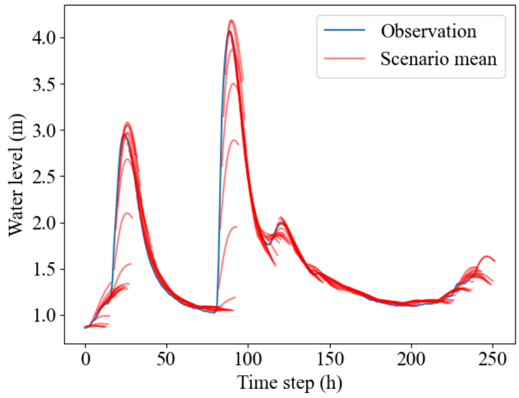


(d) MH water level for event 2

Figure 7: Hydrography with the mean of the generated scenario.

# Authors' Response to Reviewer 3

# Authors' Response to Reviewer 4

**General Comments.** This study focuses on generating reservoir inflow scenarios using a Bayesian Neural Network and evaluating methods for reducing these scenarios. This topic is important for practical water resources applications, particularly in reservoir flood control optimization. The revised manuscript is meaningful and contributes valuable insights to the field, with results that adequately support its intended contribution. The paper can be recommended for publication after the following revisions:

**Response:** We thank to your positive and constructive feedback.

We have carefully considered and addressed all comments you pointed out. The remarks have allowed us to improve the quality of the manuscript.

**Comment 1**

The Abstract is too long and includes excessive technical detail that would be more appropriate in the main text. There is redundancy, particularly around scenario generation and reduction concepts, which are repeated several times. Consider reducing redundancy and shortening it to under 300 words for clarity and conciseness.

**Response:** Thank you for this constructive feedback.

To enhance legibility and clarity, we completely rewrote the abstract following your recommendations, which is now more concise and less redundant, as follows:

**Revised Abstract**: Dealing with uncertainty in predicted inflows presents a major challenge in optimal reservoir flood control. Scenario-based stochastic control approaches address this by generating multiple inflow time series from probabilistic models, each representing a possible future with associated likelihoods.

However, using too many scenarios increases computational complexity, while too few may compromise representativeness. Although the two critical steps of scenario generation and reduction have been extensively explored in other fields, their application to reservoir inflow dynamics remains limited. This study develops and applies a probabilistic data-driven model—specifically, a Bayesian Neural Network (BNN), for scenario generation. The BNN effectively captures temporal dependencies in inflow time series and achieves high short-term accuracy, as measured by the Nash–Sutcliffe Efficiency Coefficient (NSE) and Root Mean Squared Error (RMSE), though performance declines over longer horizons.

For scenario reduction, four distance measures widely used in other domains, i.e., the Manhattan, Euclidean, Wasserstein, and energy distances, are evaluated. Experimental results show that the energy distance best preserves the statistical properties of the full scenario set, followed by the Manhattan and Euclidean distances. However, in terms of retaining extreme inflow scenarios, which are critical for flood control, the Manhattan and Euclidean distances outperform others based on a custom index measuring the envelope size of the original scenario set using the $l_1$-norm. In terms of computational efficiency of scenario reduction approaches, the energy distance is the most expensive (quadratic in $m$, the number of reduced scenarios), while the Wasserstein scales linearly. In the examples used, reduced sets are shown to adequately capture extremes when the number of scenarios $m \geq 30$. Considering the trade-off between preserving extremes and computational cost, the Manhattan and Euclidean distances with $m = 30$ are recommended as a practical choice for reservoir inflow scenario reduction.

**Comment 2**

The manuscript uses “NSC” as an abbreviation for Nash-Sutcliffe Efficiency, which is not standard. Please use the widely accepted abbreviation “NSE” for clarity and consistency with the literature. Consider citing some updated papers and incorporating the performance metrics discussed in these studies to strengthen the evaluation of model performance.

**Response:** Thank you for this valuable comment.

We totally agree with your recommendation. We have changed "NSC" to "NSE" all over the manuscript, for example, in line 297. Moreover, some recent papers adopting NSE and RMSE as performance metrics have been added, such as Melsen et al. (2025), Vishwakarma et al. (2025), and Ma et al. (2025), in addition to an original reference for NSE, Nash et al. (1970).

Regarding incorporating more performance metrics to strengthen the evaluation of model performance, we kindly ask for your understanding that the performance metrics generally adopted in previous relevant papers, which are the Mean Absolute Error (MAE) and Mean Absolute Percentage Error (MAPE), except for NSE and RMSE, are not likely to present different ideas to evaluate the performance of our model. This is because it seems clear that our BNN models can predict inflows quite accurately when the inflow is yet low, and MAE and MAPE focus more on low inflows compared to RMSE, which concentrates more on high inflows, such as peaks.

To clarify this, we have added a sentence, as follows:

**Added text L299-301**: In reservoir flood control, reducing significant deviations, such as peak inflows, is more important than minimizing minor discrepancies. Therefore, RMSE appears to be a suitable metric, as it places greater emphasis on larger errors.

**Comment 3**

The spelling and capitalization of method names and their abbreviations should be carefully reviewed to ensure consistency and alignment with common literature standards. Abbreviations should be defined in full at their first occurrence, followed by the abbreviation in parentheses. Subsequent mentions should use only the abbreviation.

**Response:** Thank you for this constructive comment.

We identified several inconsistencies in the use of abbreviations throughout the manuscript. We have carefully reviewed the entire text and corrected these issues, ensuring that only abbreviations are used after their first occurrence. For example, Bayesian Neural Network (BNN) is shortened to BNN in line 100 and in the caption of Fig 3, Korea Water Resources Public Corporation is shortened to K-water in line 240, and Tree-structured Parzen Estimator (TPE) is shortened to TPE in line 278. Additionally, we found that the full name of TPE we presented was not aligned with common literature standards, thus, we have revised it from the Tree Parzen Estimator (TPE) to Tree-structured Parzen Estimator (TPE) in line 276.

In the Introduction section, we also found capitalization inconsistencies in abbreviation definitions, as you pointed out. We have revised them as follows: autoregressive-moving average (ARMA) → AutoRegressive-Moving Average (ARMA); periodic ARMA (PARMA) → Periodic ARMA (PARMA); autoregressive integrated moving average (ARIMA) → AutoRegressive Integrated Moving Average (ARIMA) in lines 14–16; Long short-term memory (LSTM) → Long Short-Term Memory (LSTM) in line 31.

In general, the plural form of a neural network abbreviation is used when referring to such models collectively. Therefore, we have converted them into the plural form when referring to general model categories, for example, Generative Adversarial Networks (GANs), Deep Neural Networks (DNNs), and Bayesian Neural Networks (BNNs) in lines 31-34.

Finally, we added the full name of ReLU at its first occurrence, which was not defined in

the original manuscript, in lines 294-295.

### Comment 4

The manuscript, especially the Abstract, frequently uses the active voice (e.g., “we develop,” “we evaluate”). It is generally recommended to use the passive voice to improve objectivity and better align with conventional academic style.

**Response:** Thank you for your valuable feedback regarding the use of active voice in the manuscript, particularly in the Abstract.

We appreciate your suggestion to adopt a more passive voice to enhance objectivity and align with conventional academic style. Accordingly, we have revised the Abstract (see response to Comment 1) and other relevant sections to incorporate a more balanced use of passive constructions while maintaining clarity and readability.

**Revised text L124-126**: By randomly deactivating some nodes (dropout) and repeating random dropouts (Monte-Carlo simulation), multiple estimated outputs, i.e., uncertain scenarios, are generated.

**Revised text L210-212**: The distances between two scenarios to sort scenarios, i.e., clustering, and pick one representative scenario in each cluster can be utilized.

**Revised text L212-214**: In contrast, the similarity between the original scenario set and the reduced set can be compared by the Wasserstein or energy distance (Heitsch et al. 2007; Ziel 2021).

**Revised text L214-217**: Scenarios can be added one by one to the initially empty reduced set, each selected to minimize the distance between the two distributions (forward selection). On the other hand, scenarios can be removed from the reduced

set, which initially contains all original scenarios (backward selection).

**Revised text L220-222**: When the forward or backward selection method is applied using the Manhattan or Euclidean distance, it is clear that only the scenario closest to the mean is selected at each step.

**Revised text L275-277**: In this research, the Tree-structured Parzen Estimator (TPE) algorithm (Bergstra et al. 2011; Watanabe 2023) is employed. It is a popular and widely validated method in hyperparameter optimization.

**Revised text 287-288**: This algorithm is implemented using Optuna (Akiba et al. 2019), a Python library.

**Revised text L367-368**: To evaluate this, estimating a scenario envelope and comparing its size with the envelope size of the original scenarios is necessary.

**Revised text L379-381**: This envelope volume, i.e., the span of flood risk in the scenario set, can be used as a metric of goodness for assessing the reduced scenario set.

**Revised text L403-404**: The mean and standard deviation of the original and reduced scenarios are compared, as shown in Fig 10.

**Revised text L454-456**: The MC dropout BNN model is found to effectively generate uncertain inflow scenarios, with reasonable performance demonstrated in terms of RMSE and NSE.

**Revised text L477-478**: The advantages and disadvantages of existing scenario reduction methodologies, particularly from the perspective of distance measures,

are evaluated.

However, we have retained some active sentences beginning with "we" when it is important to clearly indicate that the actions were performed or decisions were made by the authors in this study, for example,

## Comment 5

The Introduction lacks a clear, concise statement of the study's novelty and specific contribution within reservoir inflow scenario generation and reduction.

**Response:** Thank you for this valuable comment regarding the need for a clearer and more concise statement of the study's novelty and specific contributions.

In response, we have revised the final part of the Introduction to explicitly highlight the research gap, the novelty of our work, and its main contributions within the context of reservoir inflow scenario generation and reduction. In addition, the contributions are now presented as a structured numbered list for clarity, as follows:

**Revised text L78-98**: To the best of our knowledge, this is the first systematic study that integrates the verification of a probabilistic data-driven approach for reservoir inflow scenario generation with a systematic, quantitative evaluation of multiple scenario reduction techniques. While scenario generation and reduction have been investigated in other domains, no prior research has jointly examined these methodologies in the context of reservoir inflow with explicit consideration of flood events. In this study, two aspects are addressed: (i) statistical representativeness of reduced scenario sets, which has been widely studied in the literature, and (ii) preservation of extreme inflow events, which is critical for reservoir flood control yet remains largely underexplored.

The specific contributions of this research are as follows:

1. **Developing and validating** a Bayesian Neural Network–based method for generating realistic reservoir inflow scenarios that accurately capture temporal dependence in hydrological time series.

2. **Conducting a systematic comparison** of four widely used distance measures—the Manhattan, Euclidean, Wasserstein, and energy distances—for hydrological scenario reduction, assessed using metrics tailored to both statistical fidelity and extreme-event preservation.

3. **Providing guidance** on selecting a practical number of reduced scenarios and the most appropriate distance measures for reservoir flood control applications, considering the trade-offs between statistical accuracy, computational efficiency, and the preservation of extreme scenarios.

**Comment 6**

The literature review is too detailed and occasionally unfocused. Consider condensing the literature review by focusing on the most relevant studies and directly linking them to the research gap addressed in this work. Consider including recent studies on sustainable flood risk management to better connect the practical relevance of inflow uncertainty with broader flood management frameworks.

**Response:** Thank you for this valuable comment.

We have revised the literature review part in the Introduction to make it concise and link it to the research gap directly, as you pointed out, as follows:

**Revised text L13-72**: Scenario generation for hydrological variables such as reservoir inflow or water level has traditionally been based on statistical models, including AutoRegressive–Moving Average (ARMA), Periodic ARMA (PARMA), and

AutoRegressive Integrated Moving Average (ARIMA), or directly from historical observation data (Dires et al. 2023). Common approaches include adding random noise drawn from a normal distribution to historical series (H. Li et al. 2018) or increasing the standard deviation of this noise over longer horizons to reflect growing uncertainty (Koo et al. 2024). Historical records can be treated as scenarios with equal probability (B. Xu et al. 2017), or scenarios can be generated by perturbing ARMA predictions with uniformly distributed random values (Haddad et al. 2014). Another widely used method is the scenario tree (Kaut et al. 2003), where historical data are discretized into a branching structure. Nonparametric approaches for generating scenario trees have been applied to stochastic hydroelectric optimization (Pflug et al. 2016; Séguin, Fleten, et al. 2017; Séguin, Audet, et al. 2017). However, in hydrological applications, scenario trees are less common because multi-step inflow scenarios are required for reservoir flood control, leading to high dimensionality and computational intractability in optimal control approaches.

In recent years, data-driven models have been applied for scenario generation in various fields, such as autonomous driving (Ding et al. 2023) and power systems (Sideratos et al. 2012; Vagropoulos et al. 2016). Deep learning architectures, including Bayesian Networks, Convolutional Neural Networks (CNNs), Long Short-Term Memory (LSTM) networks, and Generative Adversarial Networks (GANs), have been explored for generating realistic scenarios. However, most have been used in deterministic prediction settings. Probabilistic approaches such as Quantile Regression Deep Neural Networks (QRDNNs) can capture the conditional distribution of target variables (Heijden et al. 2022; Heijden et al. 2025). Still, when applied to multi-step problems, it is not possible to ensure the preservation of temporal dependence. By contrast, probabilistic models such as Gaussian Processes and Bayesian Neural Networks (BNNs) directly estimate parameter distributions from data (Jospin et al. 2022; Gal et al. 2016), making them well-suited to generating hydrological inflow scenarios that maintain temporal dependence in time series

data.
While a large number of scenarios better capture hydrological uncertainty, excessive scenarios make stochastic optimization computationally intractable (Dvorkin et al. 2014). Scenario reduction is a technique to manage the computational efficiency without losing the properties of the original scenario set Germscheid et al. 2023. A k-means clustering algorithm was applied to generate a scenario tree (D. Xu et al. 2012), while forward selection and backward reduction methods based on the Wasserstein distance were proposed (Dupačová et al. 2003). Clustering is one of the most widely used scenario reduction methods. In a stochastic optimization problem for power demand response scheduling, k-means clustering achieved the highest risk aversion in terms of cost and risk among several methods, including probability distance–based reduction (Germscheid et al. 2024; Heitsch et al. 2003). For the stochastic unit commitment problem, fast forward selection based on the Wasserstein distance was found to be more effective in terms of operational cost compared with k-means using the Euclidean distance, backward reduction, and importance–sampling–based reduction (Dvorkin et al. 2014).
Despite the advances in scenario reduction techniques in various applications that use time series forecasting, significant gaps remain in the context of hydrological scenario reduction. Compared to the Euclidean distance embedded in the k-means clustering, which is relatively extensively studied, and the Wasserstein distance, which is most widely used for scenario reduction in various fields (Ziel 2021; Rujeerapaiboon et al. 2022), the Manhattan distance and energy distance metrics have been less frequently applied. In particular, to the best of the authors' knowledge, few studies have investigated the suitability of distance measures for hydrological scenario reduction. For example, even though capturing extreme events is crucial for flood control due to their significant impact on hydrological flood conditions, it seems that the most effective reduction technique for preserving extreme events from the original scenarios has not been fully explored. In addition,

the effectiveness of reduction methods is commonly assessed by means of numerical experiments applied to stochastic optimization problems. (Germscheid et al. 2024; Zhang et al. 2023; Dvorkin et al. 2014; Sharma et al. 2013). However, performance may depend on the experimental sample, and separating the influence of the scenarios from the stochastic optimization approaches is rather difficult. Accordingly, it is essential to separately examine how much the reduced scenario set preserves the characteristics of the original scenario and how well it fits the characteristics of the flood control (Horejšová et al. 2020; Hu et al. 2019). In this paper, the performance of the reduction approach is examined by only examining the characteristics of the scenario sets and not their role within a closed-loop stochastic control setting.

In addition, a paragraph has been added to describe the practical contributions of this study to reservoir operation.

**Revised text L73-77**: From a broader perspective, sustainable flood management increasingly emphasizes integrating inflow uncertainty into reservoir control strategies (Huang et al. 2022; X. Li et al. 2010). Scenario generation and reduction methods that retain both statistical fidelity and extreme-event coverage are, therefore, not only methodologically important but also directly relevant to the robustness and resilience of real-world flood control operations.

To clarify the connection of the literature review to our research, we have modified the start paragraph of the Introduction, as follows:

**Revised text L2-12**: Model-based optimal reservoir flood control, typically implemented via model predictive control (MPC) (Koo et al. 2024), requires predictive regulation of discharge based on forecasts of inflows. Uncertainty in the control operation is therefore mainly due to uncertainty in the prediction of inflows into a reservoir (Zhao et al. 2011), which are driven by metrological uncertainties. The inflow uncertainty in MPC can be represented as a limited number of feasible

future scenarios (Séguin, Audet, et al. 2017). Therefore, scenario generation approaches aim to generate discrete time-series that can represent this uncertainty. In the case of stochastic optimization-based MPC approaches, the number of scenarios has a profound impact on the computational complexity; therefore, it is desirable to reduce the number of scenarios while at the same time preserving enough scenarios that 'sufficiently' represent the inflow uncertainty (Löhndorf 2016; Ziel 2021).

### Comment 7

Add a flowchart in the Method section to visually summarize the proposed scenario generation and reduction steps for clearer understanding.

**Response:** Thank you for this constructive comment.

We totally agree with your comment and believe that a flowchart can improve the clarity and readability of the manuscript. Therefore, we added a figure illustrating our methodologies and the explanatory sentences at the end of the Method section, as follows:

**Added text L229-233**: The method presented in this section is illustrated in Fig 1. Scenario generation is performed using MC dropout BNN, and the resulting (very large number of) scenarios are reduced using four distance measures. These reduced scenarios are then evaluated based on criteria that best replicate the original generated scenarios. The details of this process are provided in the following sections.

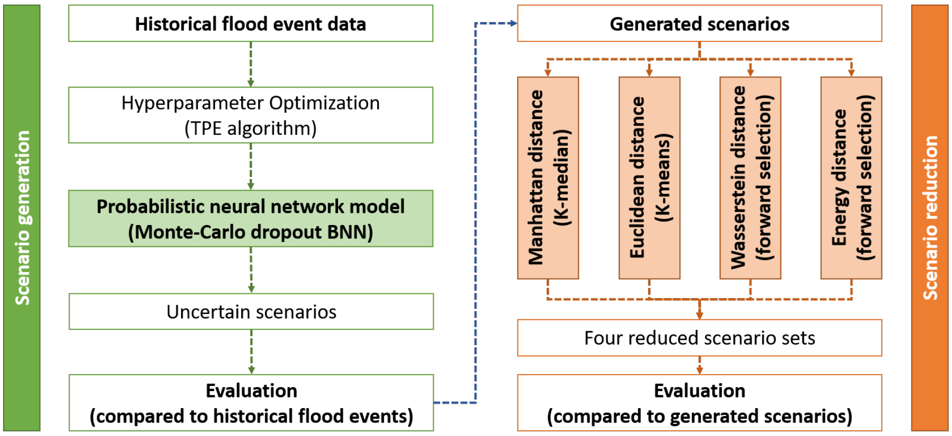


Figure 1: Flowchart of the study framework, comprising scenario generation and scenario reduction. The methods are applied to reservoir flood inflow time series to assess their practical applicability.

## Comment 8

Some sentences throughout the manuscript are overly long or contain multiple ideas, which affect clarity and flow. It is recommended to simplify these sentences by breaking them into shorter, more focused statements.

**Response:** Thank you for this constructive comment.

In response, we have carefully reviewed the manuscript and revised overly long sentences by breaking them into shorter ones or by removing redundant phrases, more focused statements. We believe these changes improve readability and flow while preserving the original meaning.

**Revised text L124-127**: By randomly deactivating some nodes (dropout) and repeating random dropouts (Monte-Carlo simulation), multiple estimated outputs are generated. These multiple outputs are uncertain scenarios.

**Revised text L157-158**: K-median is more effective for data with many outliers and asymmetric distributions due to the $l_1$-norm.

**Revised text L164-166**: The continuous scenario reduction offers flexibility to find reduced sets from infinitely many options from a continuous set. However, it also has issues concerning the validity of the new scenarios (Rujeerapaiboon et al. 2022).

**Revised text L214-217**: Scenarios can be added one by one to the initially empty reduced set, each selected to minimize the distance between the two distributions (forward selection). On the other hand, scenarios can be removed from the reduced set, which initially contains all original scenarios (backward selection).

**Revised text L275-277**: In this research, the Tree-structured Parzen Estimator (TPE) algorithm Bergstra et al. 2011; Watanabe 2023 is employed. It is a popular and widely validated method in hyperparameter optimization.

**Revised text L209-301**: In reservoir flood control, reducing significant deviations, such as peak inflows, is more important than minimizing minor discrepancies. Therefore, RMSE appears to be a suitable metric, as it places greater emphasis on larger errors.

**Revised text L353-354**: Therefore, we examine three representative points from each event (case 1&4: increasing, case 2&5: decreasing, and case 3&6: stable), as shown in Fig 7.

**Revised text L374-375**: These represent the flood risk space as a function of reservoir inflow and are of critical importance.

**Revised text L375-379**: Moreover, in the context of short-term flood scenarios, predicting the total inflow volume could be more important than precisely estimating each element of the inflow time series. The $l_1$-norm of each scenario represents

the total inflow volume, due to the fact that all inflow elements are positive values. Therefore, the use of the $l_1$-norm is reasonable.

**Revised text L392-397**: It is worth noting that this metric, $|\text{env}(Q)|$, does not inherently favor the Manhattan or Euclidean distances. Although it employs the $l_1$-norm to estimate total inflow volume, this use is conceptually distinct from how the Manhattan and Euclidean distances are applied in scenario reduction, and so the performance differences are not a priori expected when comparing all four distance measures.

**Revised text L454-459**: The MC dropout BNN model is found to effectively generate uncertain inflow scenarios, with reasonable performance demonstrated in terms of RMSE and NSE. Without explicitly defining temporal correlations between elements in a scenario, it reproduces the characteristic behavior of hydrological prediction, namely the degradation of performance with increasing prediction horizon (Fig 4).

**Revised text L494-496**: No substantial differences are observed between the Manhattan and Euclidean distances in terms of both statistical properties and extreme scenario preservation.

## Comment 9

Consider briefly comparing the findings with similar recent studies to better highlight the methodological contributions and contextual novelty of this work.

**Response:** Thank you for this constructive comment.

We appreciate the suggestion to compare our findings with similar recent studies. A

comprehensive literature review did not identify research directly relevant to reservoir flood control. We believe this gap itself reflects the novelty of our work.

Nonetheless, we have added brief comparisons with the most closely related studies from other domains, such as electricity demand and price forecasting, where similar reduction techniques (e.g., the Wasserstein and energy distance) were evaluated. While these studies did not address reservoir flood control or extreme-event preservation, the methodological parallels allow us to highlight the unique aspects of our framework, particularly our inclusion of a tailored metric for extreme-event coverage and our focus on the trade-off between computational efficiency and representativeness in a flood control context. We have added a paragraph to describe it in the Discussion section, as follows:

> **Added text L514-530**: Although no prior work has directly evaluated scenario generation and reduction methodologies for reservoir flood control, a similar methodological framework has been applied in the domain of electricity demand and price forecasting (Ziel 2021). In the study, energy-distance–based scenario reduction has been shown to preserve distributional properties more effectively than the widely used Wasserstein distance, albeit with higher computational cost due to its quadratic programming formulation. Comparable patterns are observed in our study, where the energy distance achieves the highest preservation of statistical properties in hydrological scenarios. However, for flood control, a non-statistical metric that measures the envelope of scenarios as a possible inflow volume metric has been introduced. Therefore, it is important to make a distinction in the performance of scenario reduction approaches based on the relevant metric tailored to the application (e.g., flood risk is related to total inflow volume). In our example, this reveals that the Manhattan and Euclidean distances outperform in retaining extreme inflow event properties, particularly for small $m$. Furthermore, by explicitly assessing computational feasibility under flood control time constraints, this study shows that energy distance may be impractical for fast real-time applications despite its statistical advantages—an operational consideration not addressed

in (Ziel 2021).

**Comment 10**

The Conclusion should briefly and impactfully summarize the key findings. The study's limitations are not sufficiently addressed. More explicit methodological recommendations for future research should be provided.

**Response:** We appreciate this valuable feedback.

We totally agree with your opinion. Therefore, we have revised the Conclusion entirely, reflecting your suggestion, as follows:

**Revised text L541-563**: This study demonstrated that Bayesian Neural Networks (BNNs) can effectively generate probabilistic flood scenarios while capturing temporal dependencies in inflow time series. A comparative evaluation of four distance measures, i.e., the Manhattan, Euclidean, Wasserstein, and energy distances, showed that the energy distance best preserves statistical properties, while the Manhattan and Euclidean distances are more effective for retaining extreme inflow events. Contrary to its widespread use, the Wasserstein distance offered no substantial advantage in this application.

From a practical perspective, the Manhattan and Euclidean distances provide a strong balance between preserving extreme events and maintaining computational efficiency, making them suitable for real-time reservoir flood control. The energy distance, although statistically superior, is computationally intensive and less viable for time-sensitive operations.

The main limitation of this study is that the operational importance of retaining extreme scenarios was not tested through direct integration into reservoir flood control optimization, such as stochastic MPC. In addition, the analysis was based

on a limited number of historical flood events, which may affect the generalizability of the findings. Future research should integrate the proposed scenario generation and reduction framework into real-time stochastic reservoir operation models to quantify operational benefits, explore hybrid approaches that combine the statistical strengths of the energy distance with the efficiency of the Manhattan or Euclidean distances, and assess performance using larger and more diverse flood event datasets to enhance robustness and transferability.

# References


Akiba, Takuya, Shotaro Sano, Toshihiko Yanase, Takeru Ohta, and Masanori Koyama. 2019. "Optuna: A Next-generation Hyperparameter Optimization Framework." In *Proceedings of the 25th ACM SIGKDD International Conference on Knowledge Discovery and Data Mining.*

Bergstra, James, Rémi Bardenet, Yoshua Bengio, and Balázs Kégl. 2011. "Algorithms for hyper-parameter optimization." *Advances in neural information processing systems* 24.

Ding, Wenhao, Chejian Xu, Mansur Arief, Haohong Lin, Bo Li, and Ding Zhao. 2023. "A survey on safety-critical driving scenario generation—A methodological perspective." *IEEE Transactions on Intelligent Transportation Systems.*

Dires, Firehiwot Girma, Mikael Amelin, and Getachew Bekele. 2023. "Inflow scenario generation for the ethiopian hydropower system." *Water* 15 (3): 500.

Dupačová, Jitka, Nicole Gröwe-Kuska, and Werner Römisch. 2003. "Scenario reduction in stochastic programming." *Mathematical programming* 95:493–511.

Dvorkin, Yury, Yishen Wang, Hrvoje Pandzic, and Daniel Kirschen. 2014. "Comparison of scenario reduction techniques for the stochastic unit commitment." In *2014 IEEE pes general meeting| conference & exposition,* 1–5. IEEE.

Gal, Yarin, and Zoubin Ghahramani. 2016. "Dropout as a bayesian approximation: Representing model uncertainty in deep learning." In *international conference on machine learning,* 1050–1059. PMLR.

Germscheid, Sonja HM, Alexander Mitsos, and Manuel Dahmen. 2024. "Scenario Reduction Methods for Risk-Averse Demand Response Scheduling under Price Uncertainty." In *Computer Aided Chemical Engineering,* 53:3223–3228. Elsevier.

Germscheid, Sonja HM, Fritz TC Röben, Han Sun, André Bardow, Alexander Mitsos, and Manuel Dahmen. 2023. "Demand response scheduling of copper production under short-term electricity price uncertainty." *Computers & Chemical Engineering* 178:108394.

Haddad, Omid Bozorg, Mahsa Farhangi, Elahe Fallah-Mehdipour, and Miguel A Marino. 2014. "Effects of inflow uncertainty on the performance of multireservoir systems." *Journal of Irrigation and Drainage Engineering* 140 (11): 04014035.

Heijden, Ties van der, Miguel Angel Mendoza-Lugo, Peter Palensky, Nick van de Giesen, and Edo Abraham. 2025. "Incorporating risk in operational water resources management: Probabilistic forecasting, scenario generation, and optimal control." *Water Resources Research* 61 (3): e2024WR037115.

Heijden, Ties van der, Peter Palensky, Nick van de Giesen, and Edo Abraham. 2022. "Day Ahead Market price scenario generation using a Combined Quantile Regression Deep Neural Network and a Non-parametric Bayesian Network." In *2022 IEEE International Conference on Power Systems Technology (POWERCON),* 1–5.

Heitsch, Holger, and Werner Römisch. 2003. "Scenario reduction algorithms in stochastic programming." *Computational optimization and applications* 24:187–206.

Heitsch, Holger, and Werner Römisch. 2007. "A note on scenario reduction for two-stage stochastic programs." *Operations Research Letters* 35 (6): 731–738.

Horejšová, Markéta, Sebastiano Vitali, Miloš Kopa, and Vittorio Moriggia. 2020. "Evaluation of scenario reduction algorithms with nested distance." *Computational Management Science* 17 (2): 241–275.

Hu, Jinxing, and Hongru Li. 2019. "A new clustering approach for scenario reduction in multi-stochastic variable programming." *IEEE Transactions on Power Systems* 34 (5): 3813–3825.

Huang, Xin, Bin Xu, Ping-an Zhong, Hongyi Yao, Hao Yue, Feilin Zhu, Qingwen Lu, Yu Sun, Ran Mo, Zhen Li, et al. 2022. "Robust multiobjective reservoir operation and risk decision-making model for real-time flood control coping with forecast uncertainty." *Journal of Hydrology* 605:127334.

Jospin, Laurent Valentin, Hamid Laga, Farid Boussaid, Wray Buntine, and Mohammed Bennamoun. 2022. "Hands-on Bayesian neural networks—A tutorial for deep learning users." *IEEE Computational Intelligence Magazine* 17 (2): 29–48.

Kaut, Michal, and W Stein. 2003. *Evaluation of scenario-generation methods for stochastic programming.* Humboldt-Universität zu Berlin, Mathematisch-Naturwissenschaftliche Fakultät II, Institut für Mathematik.

Koo, Ja-Ho, Edo Abraham, Andreja Jonoski, and Dimitri P Solomatine. 2024. "Balancing Operator's Risk Averseness in Model Predictive Control of a Reservoir System." *arXiv preprint arXiv:2407.04506.*

Li, He, Pan Liu, Shenglian Guo, Bo Ming, Lei Cheng, and Yanlai Zhou. 2018. "Hybrid two-stage stochastic methods using scenario-based forecasts for reservoir refill operations." *Journal of Water Resources Planning and Management* 144 (12): 04018080.

Li, Wei, Amin Kiaghadi, and Clint Dawson. 2021. “High temporal resolution rainfall–runoff modeling using long-short-term-memory (LSTM) networks.” *Neural Computing and Applications* 33 (4): 1261–1278.

Li, Xiang, Shenglian Guo, Pan Liu, and Guiya Chen. 2010. “Dynamic control of flood limited water level for reservoir operation by considering inflow uncertainty.” *Journal of hydrology* 391 (1-2): 124–132.

Liu, Darong, Wenchao Jiang, Lin Mu, and Si Wang. 2020. “Streamflow prediction using deep learning neural network: case study of Yangtze River.” *IEEE access* 8:90069–90086.

Löhndorf, Nils. 2016. “An empirical analysis of scenario generation methods for stochastic optimization.” *European Journal of Operational Research* 255 (1): 121–132.

Ma, Chunmei, Haoyu Jiao, Yonghong Hao, Tian-Chyi Jim Yeh, Junfeng Zhu, Huiqing Hao, Jiahui Lu, and Jiankang Dong. 2025. “Simulation of spring discharge using deep learning, considering the spatiotemporal variability of precipitation.” *Water Resources Research* 61 (4): e2024WR037449.

Melsen, Lieke A, Arnald Puy, Paul JJF Torfs, and Andrea Saltelli. 2025. “The rise of the Nash-Sutcliffe efficiency in hydrology.” *Hydrological Sciences Journal,* 1–12.

Nash, J Eamonn, and Jonh V Sutcliffe. 1970. “River flow forecasting through conceptual models part I—A discussion of principles.” *Journal of hydrology* 10 (3): 282–290.

Pflug, Georg Ch, and Alois Pichler. 2016. “From empirical observations to tree models for stochastic optimization: convergence properties.” *SIAM Journal on Optimization* 26 (3): 1715–1740.

Rujeerapaiboon, Napat, Kilian Schindler, Daniel Kuhn, and Wolfram Wiesemann. 2022. “Scenario reduction revisited: Fundamental limits and guarantees.” *Mathematical Programming* 191 (1): 207–242.

Séguin, Sara, Charles Audet, and Pascal Côté. 2017. "Scenario-tree modeling for stochastic short-term hydropower operations planning." *Journal of Water Resources Planning and Management* 143 (12): 04017073.

Séguin, Sara, Stein-Erik Fleten, Pascal Côté, Alois Pichler, and Charles Audet. 2017. "Stochastic short-term hydropower planning with inflow scenario trees." *European Journal of Operational Research* 259 (3): 1156–1168.

Sharma, Kailash Chand, Prerna Jain, and Rohit Bhakar. 2013. "Wind power scenario generation and reduction in stochastic programming framework." *Electric Power Components and Systems* 41 (3): 271–285.

Sideratos, George, and Nikos D Hatziargyriou. 2012. "Probabilistic wind power forecasting using radial basis function neural networks." *IEEE Transactions on Power Systems* 27 (4): 1788–1796.

Vagropoulos, Stylianos I, Evaggelos G Kardakos, Christos K Simoglou, Anastasios G Bakirtzis, and Joao PS Catalao. 2016. "ANN-based scenario generation methodology for stochastic variables of electric power systems." *Electric Power Systems Research* 134:9–18.

Vishwakarma, Dinesh Kumar, Salim Heddam, Arpit Gaur, Ravindra Kumar Tiwari, Ozgur Kisi, Anurag Malik, Chetak Bishnoi, Abed Alataway, Ahmed Z Dewidar, and Mohamed A Mattar. 2025. "Improving Streamflow Forecasting Efficiency Using Signal Decomposition Approaches." *Water Resources Management,* 1–34.

Wang, Xiaopeng, Hongpeng Hua, Fanwei Meng, Biqiong Wu, Hui Cao, and Zhengyang Tang. 2025. "Flood peak prediction of three Gorges reservoir in china: evidence from artificial intelligence and observation data." *Journal of Hydrologic Engineering* 30 (3): 04025008.

Watanabe, Shuhei. 2023. "Tree-structured Parzen estimator: Understanding its algorithm components and their roles for better empirical performance." *arXiv preprint arXiv:2304.11127.*

Xu, Bin, Scott E Boyce, Yu Zhang, Qiang Liu, Le Guo, and Ping-An Zhong. 2017. "Stochastic programming with a joint chance constraint model for reservoir refill operation considering flood risk." *Journal of Water Resources Planning and Management* 143 (1): 04016067.

Xu, Daobao, Zhiping Chen, and Li Yang. 2012. "Scenario tree generation approaches using K-means and LP moment matching methods." *Journal of Computational and Applied Mathematics* 236 (17): 4561–4579.

Zhang, Wei, Kai Wang, Alexandre Jacquillat, and Shuaian Wang. 2023. "Optimized scenario reduction: Solving large-scale stochastic programs with quality guarantees." *INFORMS Journal on Computing* 35 (4): 886–908.

Zhao, Tongtiegang, Ximing Cai, and Dawen Yang. 2011. "Effect of streamflow forecast uncertainty on real-time reservoir operation." *Advances in Water Resources* 34 (4): 495–504.

Ziel, Florian. 2021. "The energy distance for ensemble and scenario reduction." *Philosophical Transactions of the Royal Society A* 379 (2202): 20190431.
