# Supplementary material for: Comparison of scenario reduction approaches for reservoir inflow timeseries generated by a Bayesian Neural Network
Source: PLoS One. 2026 May 27;21(5):e0350095. doi: 10.1371/journal.pone.0350095 (PMC13215500; doi:10.1371/journal.pone.0350095)
Supplement: Attachment — Submitted filename: Review_response_JKoo_R3.pdf [file pone.0350095.s003.pdf]

Revision Note Introduction

Dear Editor and Reviewers,

We appreciate very much the feedback and suggestions, which we believe have allowed us to improve the quality and readability of the manuscript. Below, we explain how the comments and remarks have been addressed in the revised manuscript. To enhance the readability of this response letter, all the Reviewers' comments are typeset in blue boxes. Rephrased or added sentences from the revised manuscript are typeset in gray boxes. Note that line numbers, figures, and sections in the response refer to the updated version of the manuscript. The respective parts in the revised manuscript are also highlighted to indicate changes. To summarize, the major changes are listed as follows:

1. The introduction section has been carefully rewritten to improve readability and clearly articulate the contributions of this research.
2. The limitations of our study, particularly regarding modeling peaks using the BNN model, and the strategies to mitigate them have been presented and explained in detail.
3. Quantitative metrics, e.g., CRPS for probabilistic performance and ACF for temporal correlations, have been introduced to provide a statistical interpretation of the results.

In addition to the modifications made in response to the reviewers' comments, several sentences have been revised to improve clarity and readability, without altering their meaning.

Authors' Response to Reviewer 4

General Comments. Thank you for addressing all my previous comments clearly and convincingly. Some aspects could be further developed in future studies, but overall, the revised manuscript provides a valuable contribution to the field.

Response: Thank you for your positive and encouraging comments.

We appreciate the reviewer's recognition of the improvements made in the revised manuscript. We also acknowledge the reviewer's suggestion that several aspects could be further developed in future studies, which we have already discussed in the manuscript. We are grateful for the reviewer's constructive feedback throughout the review process, which has helped strengthen the manuscript.

Authors' Response to Reviewer 5

General Comments. The work provides a systematic way to generate and reduce probabilistic inflow scenarios using machine learning and quantitative metrics, helping reservoir managers make better flood-control decisions under uncertainty. The manuscript is technically rich and addresses a relevant hydrological challenge.

Response: We thank the reviewer for the positive overall assessment of our work.

We appreciate the acknowledgement of the manuscript's contributions to hydrological flood-control challenges. Our aim was to provide a systematic and practical evaluation, supported by a real case study, for generating and reducing probabilistic inflow scenarios under uncertainty, and we are pleased that the reviewer recognizes this contribution. We have carefully addressed all specific comments further to improve the clarity and rigor of the manuscript.

Comment 1

The introduction contains extensive citations and detailed explanations of prior work, making it dense and difficult to follow. It can be streamlined without losing scientific depth.

Response: We thank the reviewer for this helpful comment.

We agree that the previous version of the Introduction included overly detailed background descriptions and an extensive number of citations. In response, we have substantially revised and streamlined the entire Introduction as follows. The updated version focuses on the most relevant prior work, clarifies the key gaps motivating the study, and presents the research objectives more concisely. We believe these revisions improve the clarity and flow of the manuscript while preserving the necessary scientific depth.

Revised text L2-72: Model-based optimal reservoir flood control, typically implemented via model predictive control (MPC) [1], requires predictive regulation of discharge based on forecasts of inflows. Uncertainty in the control operation is therefore mainly due to uncertainty in the prediction of inflows into a reservoir [2], which are driven by metrological uncertainties. The inflow uncertainty in MPC can be represented as a limited number of feasible future scenarios [3]. Therefore, scenario generation approaches aim to generate discrete time-series that can represent this uncertainty. In the case of stochastic optimization-based MPC approaches, the number of scenarios has a profound impact on the computational complexity; therefore, it is desirable to reduce the number of scenarios while at the same time preserving enough scenarios that 'sufficiently' represent the inflow uncertainty [4, 5].

⋮

In this study, the Manhattan, Euclidean, Wasserstein, and energy distances are selected because they are widely used, computationally efficient, and conceptually representative of two major classes of scenario reduction methods, which are vector distance-based and probabilistic distribution distance-based approaches. Their efficiency and transparency make them suitable for repeated online reduction in reservoir flood-control settings, where real-time computation is essential. Although more advanced scenario reduction algorithms exist, such as hierarchical clustering or optimal-transport–based formulations, these methods ultimately rely on the same underlying distance measures used in this study. Focusing on the Manhattan, Euclidean, Wasserstein, and energy distances is therefore appropriate, as they constitute the core metrics underlying a wide range of reduction algorithms and provide a meaningful basis for evaluating the applicability of scenario reduction to reservoir inflow ensembles.

Comment 2

Contribution 1 (BNN-based scenario generation) is presented as novel, but such approaches already exist in hydrology and ML; the novelty is more incremental than claimed. Pleas clarify.

Response: Thank you for this important comment.

We agree that Bayesian Neural Networks (BNNs) themselves are not new and have been applied in hydrology and other domains. Our intention was not to claim methodological novelty in the BNN modelling approach itself. We have revised the manuscript to clarify that the contribution of this study lies in the application and systematic evaluation of a BNN-based probabilistic approach for multi-step reservoir inflow scenario generation within a unified scenario-generation–reduction framework. To our knowledge, such an integrated assessment—particularly with explicit attention to extreme-event preservation relevant to flood control—has not been previously examined. The revised text now emphasizes this incremental but practically meaningful contribution rather than presenting the BNN approach as novel, as follows:

Revised text L73-97: To the best of our knowledge, although Bayesian Neural Networks (BNNs) have been applied in some hydrological prediction studies, their use for multi-step probabilistic reservoir inflow scenario generation remains limited, and no prior work has evaluated their suitability within a broader scenario-reduction framework. The novelty of this study does not lie in introducing BNNs themselves, but rather in providing the first systematic integration of (i) probabilistic data-driven inflow scenario generation and (ii) a quantitative comparison of multiple scenario reduction techniques tailored to reservoir flood-control needs. In particular, previous research has not jointly examined these components with explicit attention to extreme-event preservation, which is operationally crucial yet rarely assessed. This study addresses two aspects that remain underexplored: (i) the statistical representativeness of reduced scenario sets and (ii) their ability to retain extreme

inflow trajectories that strongly influence reservoir flood-control decisions. The specific contributions are as follows:

1. **Applying and evaluating** a Bayesian Neural Network to generate probabilistic multi-step inflow scenarios and examine their uncertainty propagation and temporal dependence.
2. **Conducting a systematic comparison** of four widely used scenario reduction distance measures—the Manhattan, Euclidean, Wasserstein, and energy distances—using metrics designed to assess both statistical fidelity and extreme-event preservation.
3. **Providing practical guidance** on selecting an appropriate number of reduced scenarios and choosing distance measures suited for real-time reservoir flood control, considering the balance between computational efficiency, statistical quality, and the retention of extreme scenarios.

Revised text L522-524: This study established a probabilistic framework for reservoir flood control by applying Bayesian Neural Networks (BNNs) to generate inflow scenarios, verifying their applicability in capturing temporal dependencies in inflow time series.

Comment 3

There is no performance comparison with other deep learning models (such as LSTM, GRU, CNN), which weakens the justification for BNN. Authors are suggested to provide the performance comparison with other deep learning models.

Response: Thank you for this valuable comment.

We fully agree that many deep learning models, such as LSTMs, GRUs, and CNNs,

are widely used in hydrological forecasting. However, the aim of this study is not to demonstrate the superiority of a specific model, but rather to evaluate the applicability of a probabilistic Bayesian Neural Network (BNN) for generating multi-step inflow scenarios and to systematically examine how different scenario-reduction distance measures perform on such probabilistic scenario sets.

Most deep learning models mentioned by the reviewer are designed for deterministic prediction, not for producing an ensemble of probabilistic scenarios. To generate multiple scenarios using these models, additional probabilistic frameworks or model-specific extensions (e.g., MC dropout, variational layers, bootstrap ensembles, or GAN-based stochastic decoders) would be required. Implementing and validating these extensions would substantially expand the methodological scope of the paper and is beyond the intended focus of this study.

We have therefore clarified in the revised manuscript that the contribution of this work is not to compare BNNs with other deep learning architectures, but to assess how well a probabilistic BNN can generate multi-step inflow scenarios that preserve temporal dependence, and to evaluate the performance of reduction methods on these probabilistic scenario sets. We believe this framing aligns with the objectives of the paper, and we have revised the text accordingly to avoid implying that BNNs outperform other deep learning models, as follows:

> **Added text L33-38**: To clarify, extending deterministic data-driven models to produce probabilistic multi-step scenarios would require additional stochastic mechanisms, e.g., Bayesian layers, ensemble sampling, or generative components, and substantially broaden the methodological scope. Since the objective of this study is to evaluate the applicability of a probabilistic model—rather than compare different deep learning architectures—we focus on BNNs as a representative probabilistic approach.

Comment 4

The TPE algorithm is explained in textbook detail, which disrupts the methodological flow and adds complexity that could be summarized briefly.

Response: Thank you for pointing this out.

We agree that the previous description of the TPE algorithm contained unnecessary textbook-level detail and interrupted the methodological flow. In response, we have substantially shortened and simplified the explanation so that it only highlights the role of TPE in this study. The revised text now provides a concise description of TPE as a hyperparameter optimization tool and removes technical details that are not essential to the contribution of the paper, as follows. This improves readability while retaining the necessary methodological clarity.

> **Revised text L284-289**: Hyperparameters of the probabilistic inflow model are optimized using the Tree-structured Parzen Estimator (TPE) algorithm [6, 7], a widely used Bayesian optimization approach. TPE models the performance of past hyperparameter trials and proposes new candidates by favoring configurations expected to improve model accuracy. The Root Mean Square Error (RMSE) of the validation set is used as the objective function. All optimization is implemented using the Optuna library [8].

Comment 5

The use of random validation sampling for time-series prediction is problematic. Temporal data requires chronological splitting to avoid information leakage.

Response: Thank you for raising this point.

We agree that chronological validation is generally recommended in time-series prediction to avoid information leakage. However, in our study, the temporal dependency that must

be preserved exists *within each multi-step inflow scenario*, not across different timestamps in the dataset.

At each time step t, the BNN generates a complete multi-step scenario $[I_t, I_{t+1}, \ldots, I_{t+11}]$, and the temporal structure inside this vector is fully preserved during training. Because a new set of input features is provided at every time step, the model does not use any future information (i.e., one predicted at the previous time step,) when generating the scenario at each time step. Therefore, the temporal correlation relevant for prediction is internal to each generated scenario and is not affected by whether validation instances are selected randomly or chronologically. This implies that all data instances do not share information and can therefore be treated as mutually independent.

We acknowledge that a strictly chronological validation split would be methodologically ideal in a conventional sequential forecasting setup. However, adopting such a scheme in our setting would substantially reduce the number of extreme inflow events available in the training or validation datasets. For example, because two flood events are reserved for testing, only one flood event would remain for validation if six were used for training. This could lead the hyperparameter optimization process to overfit to one validation event, whereas random sampling can allow the validation set to contain a more balanced representation of both normal and extreme conditions.

In contrast, random validation sampling preserves the number of extreme-event instances available during hyperparameter optimization, and all validation instances are independent in our formulation. Since the validation dataset is used solely for hyperparameter tuning rather than for evaluating temporal forecasting performance, random validation sampling provides an efficient and appropriate approach in this context. Nevertheless, we appreciate the reviewer's attention to this issue and have clarified this point in the revised manuscript.

> **Added text L266-272**: Note that, because the model generates a complete multi-step inflow scenario at each time step using only information available at each time step, the temporal dependency that must be preserved is internal to each

scenario vector rather than across different timestamps in the dataset. Therefore, given that only a small number of flood events are available, random sampling for the validation does not induce information leakage in this setting, even though chronological validation is generally recommended in time-series prediction.

Comment 6

Although k-means, k-median, and forward selection are used, the manuscript lacks a strong justification for using these specific algorithms over more modern or optimal alternatives.

Response: Thank you for this insightful comment.

We agree that various modern clustering and optimal transport–based reduction techniques exist. In this study, however, the goal was not to identify the globally optimal reduction algorithm but to evaluate how distance measures commonly used in practice behave for hydrological inflow scenario reduction. The algorithms chosen—k-means, k-median, and fast forward selection—are among the most widely used and well-established reduction approaches in stochastic optimization, and they represent three conceptually distinct families: centroid-based clustering, medoid-based clustering, and probability-distance-based selection.

These methods were selected because they are computationally efficient, transparent, and tractable for generating reduced scenario sets at each time step of reservoir operation—a setting where real-time computation is critical. In contrast, more modern or computationally intensive alternatives (e.g., hierarchical clustering, optimal transport solvers using linear programming, or deep generative model–based compression) are significantly more expensive and less suitable for repeated online reduction of inflow ensembles.

We have added a clarification in the manuscript to better explain this rationale and to emphasize that the aim of this study is to assess the behavior of commonly adopted,

computationally tractable reduction techniques for reservoir flood-control applications.

Added text L60-72: In this study, the Manhattan, Euclidean, Wasserstein, and energy distances are selected because they are widely used, computationally efficient, and conceptually representative of two major classes of scenario reduction methods, which are vector distance-based and probabilistic distribution distance-based approaches. Their efficiency and transparency make them suitable for repeated online reduction in reservoir flood-control settings, where real-time computation is essential. Although more advanced scenario reduction algorithms exist, such as hierarchical clustering or entropy-regularized optimal transport algorithms, these methods ultimately rely on the same underlying distance measures used in this study. Focusing on the Manhattan, Euclidean, Wasserstein, and energy distances is therefore appropriate, as they constitute the core metrics underlying a wide range of reduction algorithms and provide a meaningful basis for evaluating the applicability of scenario reduction to reservoir inflow ensembles.

Comment 7

Only 9 flood events exist in the dataset. This large imbalance between normal and extreme flows severely limits peak-performance learning, yet the issue is not addressed with corrective techniques (e.g., resampling, extreme-event augmentation).

Response: Thank you for this important comment.

We agree that the limited number of historical flood events is a constraint and that, in principle, corrective techniques such as resampling or extreme-event augmentation could be considered. However, in the context of multi-step inflow scenario generation, both training and testing rely on continuous time-series data, making conventional resampling difficult and problematic.

Regarding data augmentation, most existing approaches (e.g., synthetic generation using

ANNs, BNNs, autoencoders, or generative models) require large and diverse datasets to reliably reproduce the statistical characteristics of extreme hydrological events. Designing augmentation methods specifically for rare extreme inflows is itself an open research challenge in hydrology and lies beyond the scope of this study.

Importantly, although the historical record contains only nine flood events, the probabilistic BNN generates many stochastic inflow scenarios at each time step. These scenarios span a range of possible values that can exceed, approach, or approximate observed peaks, as demonstrated in Fig 6. Thus, even with limited peak observations, the scenario ensemble can still represent peak-like behavior.

While we acknowledge that the scarcity of extremes may limit peak-performance learning, our primary objective is to assess how probabilistic scenario generation and reduction methods behave under realistic data availability conditions typical of reservoir inflow datasets. We have added a clarification in the manuscript to explicitly discuss this limitation and its implications.

> **Added text L263-265**: Despite the limited number of observed extremes, the probabilistic scenario ensemble can still cover peak-like inflow behavior through stochastic variability.

Comment 8

> The model underpredicts flood peaks - a major practical limitation for reservoir flood control -but no strategy to mitigate this is proposed.

Response: We thank the reviewer for highlighting this critical practical issue.

We acknowledge that the mean of the generated scenarios tends to underpredict flood peaks due to the scarcity of extreme flood events in the training data.

We would like to clarify that our primary mitigation strategy lies in the probabilistic

nature of the BNN and the scenario-based control framework itself, rather than relying on a deterministic point forecast (i.e., the mean).

However, we fully agree with the reviewer's perspective on the model performance. Even in a probabilistic framework, it would be far more desirable if the scenario mean could accurately capture the peak inflow, as this would imply a higher degree of model precision and reduced predictive uncertainty. While the current model relies on the ensemble spread to capture extremes due to data limitations, improving the accuracy of the mean prediction remains a key objective for our future model refinement. In addition, it is desirable to describe not only this limitation but also potential methodologies to mitigate it.

To fundamentally address this, advanced modeling techniques such as Transfer Learning (to leverage hydrological patterns from data-rich basins) or Regime-Switching Multi-Model Frameworks (which utilize specialized sub-models for high-flow and low-flow regimes) could be employed. However, developing and optimizing these complex predictive architectures falls outside the scope of this study. Therefore, we have explicitly discussed these potential architectural enhancements as promising directions for future research in the revised manuscript, as follows.

Added text L538-543: Consequently, the model tends to underpredict the mean of extreme events. While this risk is mitigated in our framework by utilizing the probabilistic upper bounds, future studies could aim to improve the mean accuracy. Potential approaches include employing transfer learning [**xu2023deep**] or regime-switching multi-model frameworks to explicitly capture high-flow dynamics [**mari2023deep**]. However, such architectural enhancements are outside the scope of this study.

Comment 9

Since the model is probabilistic, metrics such as CRPS, prediction interval coverage, or quantile errors should be included.

Response: We really appreciate this reviewer's suggestion.

Following your recommendation, we calculated the Continuous Ranked Probability Score (CRPS), Prediction Interval Coverage Probability (PICP), and Quantile Loss using the empirical distribution of the 1,000 scenarios. We analyzed these metrics for both the 95% confidence interval and the full ensemble range (Min-Max, denoted as 100%), across prediction horizons and for the total inflow volume (l_1-norm).

The results yielded three key insights:

- Temporal Consistency: CRPS and Quantile Loss consistently increase as the prediction horizon extends (e.g., CRPS from 102.5 to 216.9), accurately reflecting the accumulation of uncertainty over time.
- Under-dispersion in Extremes: The PICP for the 95% interval is relatively low (approx. 0.33–0.43). This indicates that the model is under-dispersed during extreme flood events, primarily due to the dominance of low-flow data in the training set.
- Operational Reliability: However, when considering the full ensemble range (Min-Max), the coverage improves significantly (approx. 0.65). Crucially, for the total inflow volume—which is the critical factor for reservoir flood control—the Min-Max PICP reaches 0.85.

This confirms that while the model's core distribution is narrow, the extreme scenarios (upper/lower bounds) successfully capture the risk. This finding strongly validates our proposed scenario reduction strategy, which prioritizes preserving the Envelope Size (l_1-norm) to ensure these safety-critical boundaries are retained for flood control operations.

Additionally, we acknowledge that the probabilistic metrics, particularly the 95% PICP (approx. 0.4), indicate that the BNN results are not statistically perfect in terms

of uncertainty calibration. However, we argue that statistical imperfection does not necessarily preclude practical utility. The definitive assessment of whether the model is applicable for practical reservoir operation should be determined by its performance in downstream applications, specifically optimal control simulations. Given that the model successfully captures the total inflow volume (PICP 100% ≈ 0.85) coverage, we believe the current framework demonstrates sufficient potential for operational use. We have clarified in the revised manuscript that the observed limitations are primarily driven by the scarcity of high-inflow training data. Furthermore, we have expanded the Discussion section to suggest specific methodologies to overcome this barrier in future research, such as transfer learning or regime-switching multi-model frameworks.

We are truly grateful for this constructive feedback. It has significantly strengthened the validity of this study and inspired ideas for our follow-up research.

Table 1: Probabilistic performance metrics averaged over flood events. '100%' denotes metrics calculated using the minimum and maximum of the generated scenarios.

Time (h)	PICP(95%)	PICP(100%)	CRPS(m^3/s)	Q-Loss(95%)
1	0.43	0.64	102.6	46.1
6	0.39	0.65	138.8	72.6
12	0.34	0.61	216.9	134.1
Average	**0.39**	**0.64**	150.3	78.4
Total Volume(l_1)	0.54	**0.85**	1282.7	757.8

Added text L341-355: In addition to deterministic metrics and graphical analysis, we evaluated the probabilistic performance of the BNN model using the Continuous Ranked Probability Score (CRPS), Prediction Interval Coverage Probability (PICP), and Quantile Loss, as summarized in Table 1. The CRPS and Quantile Loss consistently increase as the prediction horizon extends, correctly reflecting the natural accumulation of uncertainty over time. The PICP for the 95% confidence

interval averages around 0.39, which is lower than the nominal level. This indicates that the model is under-dispersed, likely due to the dominance of low-flow data in the training set, which biases the model toward narrower uncertainty bounds. However, the operational reliability improves significantly when considering the full scenario range. Most crucially, for the total inflow volume (l_1-norm), which is a critical factor for reservoir flood control under short-term forecasts, the Min-Max PICP reaches approximately 0.85. This finding highlights again that the extreme scenarios (upper/lower bounds) successfully capture the operational risk space and the importance of preserving the envelope size in scenario reduction.

Revised text in Abstract: While the model exhibits limitations in predicting peak inflows due to data scarcity, it effectively captures temporal dependencies in inflow time series and achieves high short-term accuracy, as measured by the Nash–Sutcliffe Efficiency Coefficient (NSE) and Root Mean Squared Error (RMSE), though performance declines over longer horizons.

Added text L551-556: Despite these limitations, our results demonstrate that the actual peak inflow is successfully captured under or near the upper envelope. This observation underscores the critical need to capture the full spread of uncertainty rather than relying solely on the mean. Consequently, this validates our specific evaluation criteria for scenario reduction: whether a given method can effectively preserve these extreme scenarios (the envelope) to ensure that the operational risk space is maintained (Fig 6).

Comment 10

The envelope size used to evaluate extreme-event preservation is intuitive but not widely established in literature; more justification is needed.

Response: We appreciate this comment.

We acknowledge that the envelope size metric is a custom index. However, we introduced this metric to address a specific gap in standard statistical measures regarding reservoir flood control.

In reservoir operation, the total inflow volume is often more critical than the instantaneous peak for determining storage capacity in the case of short-term inflow forecasts. The l_1-norm of an inflow vector directly represents this total volume

Conventional metrics, e.g., Kolmogorov-Smirnov distance and/or moment-based measures, focus on minimizing the distance between probability distributions. Consequently, they often treat low-probability extreme scenarios as statistical outliers to be discarded. However, for flood control, the spread between the maximum and minimum total volumes, i.e., the envelope size here, represents the 'Risk Space' that the reservoir must manage Therefore, this metric serves as a very useful complement to standard metrics, ensuring that the reduced scenario set retains the worst-case boundaries required for dam safety. We have reinforced this justification in the Methodology section.

> **Added text L409-413**: While conventional distributional metrics prioritize the overall statistical fit and may discard rare extreme events as outliers, reservoir flood control requires explicit awareness of the worst-case boundaries. Therefore, the envelope size defined via the l_1-norm serves as an important additional metric to measure how well the reduced set preserves the operational safety margins.

In addition, to clarify the difference between the vector distance-based approach and the probabilistic distribution distance-based approach, we added some explanation, as follows:

> **Added text L432-453**: To understand why vector distance-based measures (Manhattan and Euclidean) better preserve extreme scenarios compared to probabilistic measures, one must examine the respective objective functions. Vector-distance approaches, implemented via clustering algorithms, aim to minimize the spa-

tial dispersion of data points. The objective functions for K-means (Euclidean) and K-medians (Manhattan) are defined as $J_{means} = \sum_{j=1}^{m} \sum_{y_i \in C_j} ||y_i - \mu_j||_2^2$ and $J_{median} = \sum_{j=1}^{m} \sum_{y_i \in C_j} ||y_i - \mu_j||_1$, where m is the number of reduced scenarios, C_j is the j-th cluster, and μ_j is the centroid. Although the l_1-norm of K-medians is generally less sensitive to outliers than the squared l_2-norm, both algorithms are fundamentally *space-partitioning* methods. An extreme flood scenario is geometrically distant from the central cluster. To minimize the total aggregated distance via both norms, the algorithm is likely to choose a representative centroid near these distant points; otherwise, the accumulated spatial error would remain significantly high.

In contrast, probabilistic measures like the Wasserstein distance incorporate the *probability mass* of each scenario into the cost function. Conceptually, the cost contribution of a scenario x_i relates to the transport cost weighted by its probability, as $Cost \approx \sum ||y_i - x_j|| \cdot w_i$, where w_i is the probability of the original scenario y_i. Since extreme flood events are rare, their associated probability w_i is very low (e.g., $p \approx 0$). Consequently, even if the geometric distance $||y_i - x_j||$ is large, its contribution to the total probabilistic cost is negligible. Therefore, probabilistic algorithms tend to "sacrifice" the preservation of these low-probability extremes to better fit the high-density regions near the distribution's center (the mean).

Comment 11

Correlation matrices are compared visually, but deeper statistical interpretation (e.g., lag-dependence preservation) is missing.

Response: We appreciate the reviewer's insightful suggestion to provide a deeper statistical interpretation of the correlation preservation.

We completely agree with the reviewer's comment that visual comparison alone is

insufficient to strictly validate the model's performance. To explicitly address the lack of statistical interpretation and lag-dependence analysis, we have reinforced our evaluation with two quantitative metrics. We introduced the Frobenius norm of the correlation matrix difference ($||R_{original} - R_{reduced}||_F$) as a metric for "structural divergence." This allows us to statistically interpret how well the global correlation structure is preserved beyond visual inspection. Additionally, we added the Autocorrelation Function (ACF) plots, as suggested, to specifically evaluate the preservation of temporal dependencies and memory decay over time. These additional analyses confirm that the Energy distance not only visually but also statistically outperforms other distance measures. We have incorporated these quantitative results and detailed discussions into the revised manuscript, as follows:

Added text L493-507: While Fig 11 and 12 provide visual comparisons, we further quantified the preservation of temporal dependencies by calculating the difference between the correlation matrices of the original and reduced sets. We employed the Frobenius norm of the difference between the correlation of distance measures ($||R_{original} - R_{reduced}||_F$) as a metric for structural divergence. This metric quantifies how well the model preserves the temporal correlations between times in the prediction horizon Table 2 summarizes the averages of these errors. The Energy distance yielded the smallest error (0.69), indicating the highest fidelity in preserving the complex lag-dependence structure of the original scenarios. The Wasserstein distance showed the largest deviation, confirming the visual discrepancies observed in Fig **??**. Furthermore, to explicitly evaluate lag-dependence preservation, we analyzed the Autocorrelation Function (ACF) in Fig 1. The results demonstrate that all measures seem to follow the same pattern, but with different values. The Energy distance (red) most closely tracks the original decay pattern, while the Manhattan (green) and Euclidean (blue) distances also adequately capture the correlations despite having slightly higher matrix errors.

Table 2: Frobenius norm of the difference distance measures

	Manhattan	Euclidean	Wasserstein	Energy
Frob. norm	1.066	1.072	1.138	0.692

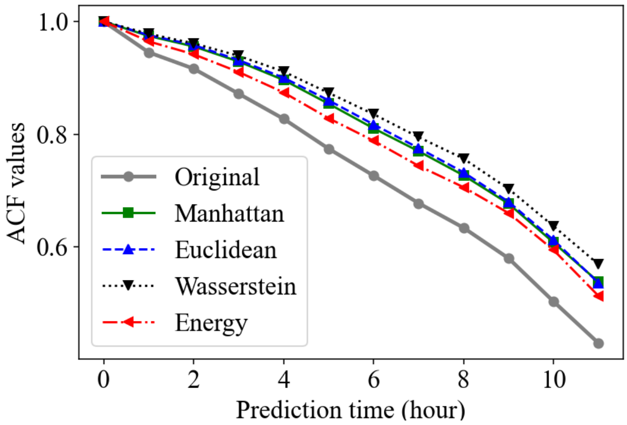

Figure 1: Comparison of temporal autocorrelation functions (ACF) across different distance measures. The energy distance (red) exhibits the highest fidelity to the original scenarios (gray), closely following its decay pattern. The Euclidean (blue) and Manhattan (green) distances also demonstrate robust performance, effectively capturing the temporal dynamics, whereas the Wasserstein distance (black) tends to overestimate temporal persistence.

References

[1] Ja-Ho Koo, Edo Abraham, Andreja Jonoski, and Dimitri P. Solomatine. “Balancing operator’s risk averseness in model predictive control for real-time reservoir flood control.” In: *Journal of Hydroinformatics* 27.4 (Mar. 2025), pp. 601–624. ISSN: 1464-7141. DOI: `10.2166/hydro.2025.019`. eprint: `https://iwaponline.com/jh/article-pdf/27/4/601/1548607/jh2025019.pdf`. URL: `https://doi.org/10.2166/hydro.2025.019`.

[2] Tongtiegang Zhao, Ximing Cai, and Dawen Yang. "Effect of streamflow forecast uncertainty on real-time reservoir operation." In: *Advances in Water Resources* 34.4 (2011), pp. 495–504.

[3] Sara Séguin, Charles Audet, and Pascal Côté. "Scenario-tree modeling for stochastic short-term hydropower operations planning." In: *Journal of Water Resources Planning and Management* 143.12 (2017), p. 04017073.

[4] Nils Löhndorf. "An empirical analysis of scenario generation methods for stochastic optimization." In: *European Journal of Operational Research* 255.1 (2016), pp. 121–132.

[5] Florian Ziel. "The energy distance for ensemble and scenario reduction." In: *Philosophical Transactions of the Royal Society A* 379.2202 (2021), p. 20190431.

[6] James Bergstra, Rémi Bardenet, Yoshua Bengio, and Balázs Kégl. "Algorithms for hyper-parameter optimization." In: *Advances in neural information processing systems* 24 (2011).

[7] Shuhei Watanabe. "Tree-structured Parzen estimator: Understanding its algorithm components and their roles for better empirical performance." In: *arXiv preprint arXiv:2304.11127* (2023).

[8] Takuya Akiba, Shotaro Sano, Toshihiko Yanase, Takeru Ohta, and Masanori Koyama. "Optuna: A Next-generation Hyperparameter Optimization Framework." In: *Proceedings of the 25th ACM SIGKDD International Conference on Knowledge Discovery and Data Mining*. 2019.
