# Supplementary material for: Comparison of scenario reduction approaches for reservoir inflow timeseries generated by a Bayesian Neural Network
Source: PLoS One. 2026 May 27;21(5):e0350095. doi: 10.1371/journal.pone.0350095 (PMC13215500; doi:10.1371/journal.pone.0350095)
Supplement: Attachment — Submitted filename: Review_response_JKoo_R4.pdf [file pone.0350095.s004.pdf]

Revision Note Introduction

Dear Editor and Reviewers,

We appreciate very much the feedback and suggestions, which we believe have allowed us to improve the quality and readability of the manuscript. Below, we explain how the comments and remarks have been addressed in the revised manuscript. To enhance the readability of this response letter, all the Reviewers' comments are typeset in blue boxes. Rephrased or added sentences from the revised manuscript are typeset in gray boxes. Note that line numbers, figures, and sections in the response refer to the updated version of the manuscript. The respective parts in the revised manuscript are also highlighted to indicate changes. To summarize, the major changes are listed as follows:

1. **Methodological Justifications:** We clarified key experimental choices, including the rationale for the training/testing data split to evaluate extreme events without data leakage, and the justification for generating 1000 initial scenarios.
2. **Technical Clarifications:** We corrected the mathematical definitions of the cluster centroids for the k-median and k-means approaches, and updated the descriptions of the computational scaling for the distance measures.
3. **Textual and Formatting Improvements:** We fixed missing section references, updated data source URLs, and corrected all typographical and grammatical errors identified by the reviewer.

In addition to the modifications made in response to the reviewers' comments, a few sentences have been revised to improve clarity and readability, without altering their meaning.

Authors' Response to Reviewer 6

General Comments. The paper addresses a relevant and timely problem in stochastic reservoir flood control: how to select a compact set of inflow scenarios that preserves both statistical fidelity and extreme-event coverage while remaining computationally feasible for real-time operation. The methodology is well-grounded, the evaluation criteria are appropriate, and the results are thoroughly discussed. The following minor issues should be addressed before the paper is accepted for publication.

Response: We would like to express our sincere gratitude to the reviewer for dedicating their time and effort to review our manuscript and for providing such encouraging and constructive feedback.

We are glad that the reviewer found our research problem relevant and timely, and that our methodology and evaluation criteria were considered well-grounded and appropriate.

We have carefully considered all the issues raised by the reviewer. We believe that incorporating these suggestions has significantly improved the clarity, accuracy, and overall quality of our manuscript. We have addressed each comment in detail and revised the manuscript accordingly. Please find our point-by-point responses below.

Comment 1

Line 5: "metrological uncertainties" should read "meteorological uncertainties." Metrology is the science of measurement, whereas meteorology is the science of weather and climate. Please correct this typo.

Response: We appreciate the reviewer pointing out this typo. We have corrected the term to "meteorological uncertainties" throughout the manuscript.

Revised text L5: Uncertainty in the control operation is therefore mainly due to uncertainty in the prediction of inflows into a reservoir [2], which are driven by meteorological uncertainties.

Comment 2

Lines 98-103: The section references in the closing paragraph of the Introduction are missing. The sentences "Section introduces the methodologies..." and "Section details the case study area..." lack their corresponding section numbers. Please correct.

Response: Thank you for this comment.

We apologize for the missing section references. This was caused by a cross-referencing compilation issue in LaTeX. We have updated the text to explicitly state 'The Method section' and 'The Results and Discussion section' to resolve this.

Revised text L98-102: The Method section introduces the methodologies for scenario generation and reduction, focusing on Monte-Carlo dropout BNN and four distance measures. The Results and Discussion section details the case study area and presents the experimental results, including evaluating a reduced scenario set based on the l_1-norm to emphasize the inclusion of extreme scenarios.

Comment 3

Line 104: The acronym "MC" is used in the subsection heading before it is defined. It is only defined later, in line 124. Please define the acronym before its first use, or spell it out in the heading ("Monte Carlo dropout BNN").

Response: Thank you for the suggestion.

We have spelled out the acronym in the subsection heading for clarity before its first use.

> **Revised text L105**: Scenario generation by Monte-Carlo dropout BNN

Comment 4

Line 173: The definition of μ_i in Eq 3 is inconsistent with the preceding text. Lines 160–162 correctly state that the k-median centroid is the element-wise median, whereas the k-means centroid is the element-wise mean. However, the caption of Eq 3 defines μ_i as "the mean of all scenarios in the cluster C_i" for both cases. For l = 1 (Manhattan distance, k-median), μ_i should be the element-wise median, not the mean. Please correct the definition to distinguish the two cases, e.g., "the centroid of cluster C_i (element-wise median for l = 1, element-wise mean for l = 2)."

Response: Thank you for the correction.

The reviewer is completely correct; our previous description inaccurately defined the centroid for the k-median approach. While revising this, we also noticed that the concluding sentence of the paragraph was mathematically imprecise. We have updated the text to accurately define the centroids for both distance metrics and to clarify the methodology as suggested.

> **Revised text L170-177**: Therefore, we modify the algorithms to select a reduction scenario from the original scenarios by identifying the one closest to each cluster's centroid (median or mean), measured by the corresponding distance measure, as follows:
>
> $$\mathbf{x}_i = \arg\min_{\mathbf{y}_j \in C_i} \|\mathbf{y}_j - \mu_i\|_l, \quad (1)$$

where $\mathbf{x}_i$ is a reduced scenario of cluster i, μ_i is the centroid of cluster C_i (element-wise median for the Manhattan distance when $l = 1$, and element-wise mean for the Euclidean distance when $l = 2$). As a result, this approach differs from conventional k-means and/or k-median clustering in that the calculated cluster centroids are not directly used as the reduced scenarios.

Comment 5

Fig 2: The URLs provided in the caption (www.data.or.kr and https://www.vworld.kr) could not be accessed. Please verify that these addresses are correct.

Response: Thank you for verifying the links.

Thank you for taking the time to verify the URLs. You are absolutely right. We have corrected the first URL to www.data.go.kr (replacing .or.kr with .go.kr). Regarding the second URL (https://www.vworld.kr), the web address is indeed correct. Unfortunately, the South Korean government restricts overseas IP access to this open geospatial data platform for security purposes. We regret the inconvenience this caused during your review and have added a short explanatory note in the figure caption to prevent future confusion for international readers.

Revised caption for Fig 2: The basin and river shape files of Korea are from the public data portal in South Korea (www.data.go.kr) and VWorld (https://www.vworld.kr; access may be restricted outside South Korea due to national security policies), respectively.

Comment 6

Lines 261-274: The training period begins in September 2012, while both test events precede this date (Event 1: August 2011, Event 2: August 2012). The rationale for starting training in September 2012 is not stated. Since data are reportedly available from 2011, a brief explanation of this choice would help readers understand whether this is a deliberate design decision (e.g., to ensure the test events are excluded from training) and its practical implications (i.e., the model is trained on data collected after the test events it is evaluated on).

Response: We sincerely appreciate the reviewer's insightful observation.

The chronological inversion—training on data from September 2012 to 2020 while testing on preceding events from August 2011 and August 2012—was indeed a deliberate design decision driven by the specific distribution of major flood events within our available dataset.

In our dataset, the most significant extreme flood events occurred at the very beginning (2011 and mid-2012) and in the middle/latter parts of the overall observation period. If we had strictly followed a conventional chronological split (e.g., training on the first 80% and testing on the last 20%), we risked either completely missing major flood dynamics in the training phase or lacking appropriate extreme events to rigorously evaluate the model during testing.

Therefore, to ensure that both the training and testing datasets included substantial flood events, we strategically partitioned the data. By isolating the 2011 and August 2012 events for testing and utilizing the September 2012–2020 period for training, we guaranteed that the model learned from severe floods while being independently evaluated on distinct, unseen historical extremes (hindcasting) without any data leakage. We have added a brief explanation of this rationale and its practical implications to the revised manuscript to provide better clarity for the readers.

Revised text L273-284: The model testing, however, is conducted on two specific flood events: a flood event in August 2011 (Event 1: 2011-08-07 to 2011-08-18, for 259 hours) and another in August 2012 (Event 2: 2012-08-12 to 2012-08-21, for 221 hours). The rationale for this chronological inversion—training on data from September 2012 to 2020 and testing on preceding events—stems from the distribution of major flood events within the available dataset. The most significant flood events occurred at the beginning and the middle of the overall observation period. To ensure that both the training and testing datasets included substantial flood dynamics, we deliberately distributed these extreme events across the sets. This specific partitioning allows the model to learn from severe floods during the 2012–2020 period while being rigorously evaluated on distinct, unseen extreme events from 2011 and early 2012, thereby preventing data leakage and effectively assessing the model's physical generalization capabilities (i.e., hindcasting).

Comment 7

Line 291: "when validation loss shows no improvement over a specified epochs" is grammatically incorrect. It should read "over a specified number of epochs."

Response: We thank the reviewer for pointing out this grammatical error.

We have corrected the phrase to "over a specified number of epochs" in the revised manuscript as suggested.

Revised text L300-302: To reduce the number of hyperparameters to be optimized, the early-stopping technique [41] is utilized, terminating the training process when validation loss shows no improvement over a specified number of epochs.

Comment 8

Line 375: The size of the original scenario set is fixed at n = 1000. This choice is not justified. A brief explanation of the rationale—whether based on convergence of the empirical distribution, a pilot sensitivity study, or computational feasibility—would strengthen the methodology.

Response: We thank the reviewer for pointing out this omission.

The choice of $n = 1000$ was determined empirically based on preliminary tests. We observed that generating 1000 scenarios was sufficient to capture the predictive uncertainty and stabilize the empirical distribution. At the same time, this number kept the computational cost of calculating the pairwise distance matrices for the subsequent scenario reduction algorithms manageable. We have added a brief explanation of this empirical rationale to the revised manuscript.

Revised text L385-390: The original scenario set Y consists of 1000 scenarios to cover possible uncertain conditions, each represented by a 12-dimensional vector. This number was determined empirically based on preliminary tests; it is large enough to capture the predictive uncertainty and stabilize the empirical distribution, yet small enough to keep the computational cost of the subsequent scenario reduction algorithms manageable. These scenarios are generated at each time step.

Comment 9

Line 498: A period is missing between "in the prediction horizon" and "Table 3." The two sentences run together without punctuation. Please insert a period after "prediction horizon."

Response: We thank the reviewer for catching this typographical error.

We have inserted the missing period after "prediction horizon" to properly separate the sentences.

> **Revised text L511-512**: This metric quantifies how well the model preserves the temporal correlations between times in the prediction horizon.

Comment 10

Lines 510-511: The claim that "the computational time increases linearly as the number of reduced scenarios... increases" is inconsistent with both the Abstract and Table 4. The Abstract correctly states that the energy distance is "quadratic in m." Table 4 confirms this: when m increases fivefold (from 10 to 50), the energy distance computation time increases approximately 24.6-fold (from 72.5 to 1787.3 s), which is consistent with quadratic scaling, not linear. Please correct line 511 to be consistent with the Abstract and the empirical data in Table 4.

Response: We greatly appreciate the reviewer for carefully reading our manuscript and catching this inconsistency.

The reviewer is absolutely correct. While the Abstract and Table 4 accurately reflect the quadratic scaling of the energy distance with respect to the number of reduced scenarios (m), the text in the Results section erroneously grouped both distance measures under a "linear" increase. We have corrected the sentence to explicitly state that the computation time scales linearly for the Wasserstein distance, but quadratically for the energy distance, ensuring full consistency with the Abstract and the empirical data.

> **Revised text L524-527**: As the number of reduced scenarios increases, the computational time increases linearly for the Wasserstein distance (from 11.8 seconds to 82.3 seconds), but scales quadratically for the energy distance (from 72.5 to 1787.3 seconds).

Comment 11

Lines 521-646 (Discussion and Conclusions): Several minor language issues were found in these sections:

- Line 572: Figures should be cited in numerical order: “Figs 8 and 9” instead of “Fig 9 and 8.”
- Line 606: “the Manhattan and Euclidean distances outperform in retaining” is missing a comparator. Please rephrase, e.g., “outperform the other measures in retaining.”
- Line 613: “can not” should be written as one word: “cannot.”
- Line 628: “other application” should read “other applications.”
- Lines 634-635: “scales much less favourably computationally with the number of scenarios in the reduced set and as such less viable for time-sensitive operations” lacks a conjugated verb for the second predicate. Please rephrase, e.g., “... and is therefore less viable for time-sensitive operations.”

Response: We sincerely appreciate the reviewer’s careful and thorough reading of our manuscript.

We have incorporated all the suggested language and grammatical corrections throughout the Discussion and Conclusions sections to improve the readability of the paper.

Revised text L585-586: For this, the Manhattan and Euclidean distances demonstrate superior performance (Fig 8 and 9).

Revised text L619-621: In our example, this reveals that the Manhattan and Euclidean distances outperform the other measures in retaining extreme inflow event properties, particularly for small m.

Revised text L626-628: Even though it has been widely applied due to its computational efficiency, this algorithm cannot guarantee the selection of the optimal reduced set.

Revised text L641-644: Contrary to its widespread use in other applications, the Wasserstein distance offered no substantial advantage in this application where temporal dependencies of the time series are important.

Revised text L647-650: The energy distance, although statistically superior, scales much less favourably computationally with the number of scenarios in the reduced set and is therefore less viable for time-sensitive operations.
